# Brain and retina in Alzheimer's disease: Pathological intersections and estimates from imaging

**DOI:** 10.1002/alz.70884

**Published:** 2025-11-27

**Authors:** M. Amin Banihashemi, Saffire H. Krance, Patrick Xiang Ji, Morgan Koo, Julie Ottoy, Richard H. Swartz, Peter J. Kertes, Christopher Hudson, Maged Goubran, Sandra E. Black

**Affiliations:** ^1^ Temerty Faculty of Medicine University of Toronto Toronto Ontario Canada; ^2^ Centre for Brain Resilience and Recovery Hurvitz Brain Sciences Program Sunnybrook Research Institute University of Toronto Toronto Ontario Canada; ^3^ Schulich School of Medicine and Dentistry University of Western Ontario London Ontario Canada; ^4^ School of Public Health Sciences University of Waterloo Waterloo Ontario Canada; ^5^ Department of Medicine Division of Neurology Sunnybrook Health Sciences Centre University of Toronto Toronto Ontario Canada; ^6^ Department of Ophthalmology and Vision Sciences University of Toronto Toronto Ontario Canada; ^7^ School of Optometry and Vision Science University of Waterloo 200 University Avenue West Waterloo Ontario Canada; ^8^ Department of Medical Biophysics University of Toronto Toronto Ontario Canada

**Keywords:** Alzheimer's disease, amyloid, blood vessels, optical coherence tomography, tau

## Abstract

Recent studies have highlighted retinal optical coherence tomography (OCT) imaging as a promising biomarker for the early detection of Alzheimer's disease (AD). This review connects AD brain pathology – particularly amyloid beta (Aβ), tau, and vascular changes – with corresponding retinal changes. Evidence suggests that abnormal Aβ and tau deposits in the retina may reflect brain pathology, though their formation mechanisms remain unclear. Retinal vascular changes may also mirror brain pathology, with recent data emerging on other co‐pathologies. Retinal thickness changes, especially in acetylcholine‐producing layers, can differentiate AD from controls, although not in early AD; however, emerging high‐resolution OCT techniques may enhance early detection. We find that correlations between retinal thickness and brain structures are often weak, and retinal vascular imaging shows promise in estimating cerebrovascular disease markers from retinal vascular changes. Novel imaging modalities (e.g., hyperspectral imaging) for detecting retinal Aβ deposits may improve early AD screening when combined with other biomarkers.

**Highlights:**

- Retinal Aβ/tau is equivocal; peripheral retinal p‐tau shows diagnostic promise.
- OCT retinal/choroid thickness diagnostic/prognostic AUC is small to medium.
- Hyperspectral imaging and electroretinography may aid early diagnosis.
- OCTA may differentiate MCI from controls, but preclinical studies are needed.
- The added value of retinal biomarkers for risk stratification remains uncertain.

## 1 INTRODUCTION

Retinal optical coherence tomography (OCT) imaging is actively used for the early detection of neurodegenerative diseases, particularly Alzheimer's disease (AD),^1^, ^2^ and their manifestations, including brain atrophy^3^, ^4^, ^5^, ^6^ and cognitive decline.^7^, ^8^, ^9^, ^10^, ^11^ Early detection of brain pathology is important, as interventions may be more effective in the early stages of dementia.^12^ Current biomarkers for early detection^13^, ^14^, ^15^ are often time‐consuming, invasive, or costly, such as brain structural magnetic resonance imaging (MRI).^15^, ^16^, ^17^ Therefore, new biomarkers are required for population‐level screening, such as retinal imaging biomarkers, which are cheaper,^18^ quicker, and more accessible.

This review aims to (1) describe the current understanding of AD pathology and its relation to the retina; (2) identify brain and retinal biomarkers that differentiate individuals across normal aging, preclinical AD, mild cognitive impairment (MCI), and AD dementia, focusing on early detection; and (3) assess the correlation of brain and retinal biomarkers, particularly imaging biomarkers, and compare their diagnostic and prognostic accuracy. We aligned our work with the proposed scale for quality assessment of narrative reviews (SANRA)^19^ and included a search strategy in the supplementary material. While several reviews have addressed aspects of this topic,^20^, ^21^, ^22^, ^23^, ^24^, ^25^, ^26^, ^27^ we aim to comprehensively discuss *post mortem* evidence, highlight underexplored areas, and evaluate the effect size of studies for clinical relevance. Most evidence is based on *post mortem* examinations and in vivo imaging in humans, with non‐human studies reviewed when human data are lacking.

## 2 Evolution, development, and anatomy

Humans have the largest primate brains, with approximately 80% of neurons in the neocortex.^28^ The primary visual cortex has neuron densities three to five times greater than other regions.^29^ Brain areas that evolved and developed earlier, such as the brainstem and spinal cord, are less vulnerable to AD pathology, whereas later‐evolving regions, including neocortical structures and those undergoing greater expansion during recent evolution, such as the temporal lobe, are more susceptible.^30^, ^31^ The visual cortex has shown less evolutionary expansion and fewer neurons containing DNA exceeding a diploid set compared to the temporal lobe and entorhinal cortex, potentially protecting it from AD pathology.^31^ Furthermore, the greater the degree of myelination of neural tracks, the less vulnerable they are to oxidative stress and AD pathology.^32^ One such region with early signs of AD pathology is the basal forebrain cholinergic system (BFCS), which modulates cognition through extensive cortical innervation.^33^ Although the BFCS is evolutionarily conserved, associated cortical areas have expanded roughly three‐fold since early hominids without a matching increase in cholinergic neurons, leaving BFCS‐cortical projections especially vulnerable to degeneration.^34^ We discuss the role of this vulnerability in AD progression further in Section 4.1.

Brain structures originate from the embryonic forebrain, or prosencephalon, which gives rise to the telencephalon (including the basal forebrain) and the diencephalon (from which the retina develops).^35^, ^36^, ^37^ Notably, cholinergic BFCS neurons arise from the ganglionic eminence of the telencephalon.^38^ Furthermore, we see shared regulatory genes responsible for embryonic forebrain development and eye development.^39^, ^40^, ^41^ These close developmental and genetic connections between the brain and eye highlight the potential of retinal imaging biomarkers to identify risk factors for brain disorders with a genetic predisposition, including AD.^42^

## 3 Alzheimer's disease pathology and co‐pathologies

**FIGURE 1:**
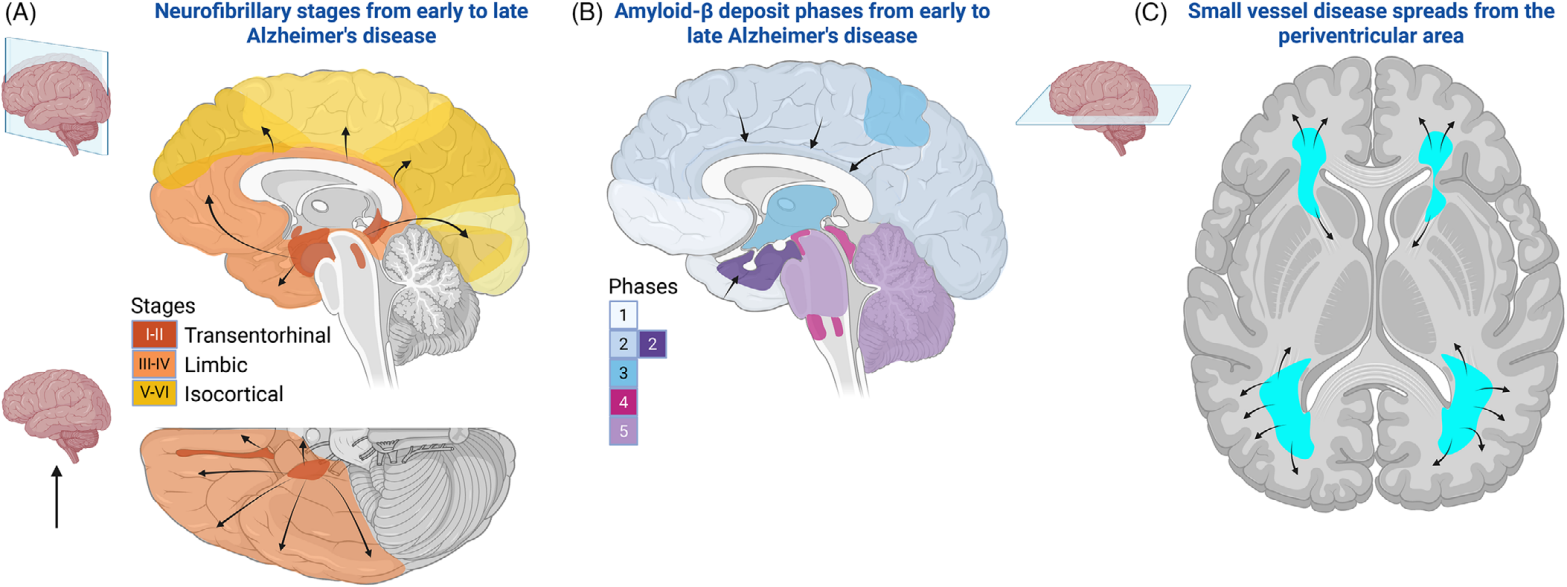
Alzheimer's disease brain pathology. (A) Tau deposits form neurofibrillary tangles, initially appearing in the entorhinal region and locus coeruleus, then spreading to subcortical nuclei and neocortex (stages I to VI).^79^, ^80^ Phase 2 is depicted with a darker blue gradient, indicating tau pathology spreading from the frontal and temporal neocortex. Purple shading emphasizes tau's characteristic spread to the entorhinal cortex during this phase, highlighting occasional amyloid plaque deposition in the amygdala, insular cortex, and cingulate cortex. (B) Amyloid beta (Aβ) deposits progress in nearly opposite direction (phases 1 to 5), as shown by autopsy and positron emission tomography imaging,^194^, ^195^ though studies linking them to retinal biomarkers are limited. (C) White matter hyperintensities start near the anterior and posterior horns of the lateral ventricles,^360^ matching areas of lower perfusion.^361^ Figure created in Biorender.

The pathogenesis of AD is primarily linked to two misfolded proteins: amyloid beta (Aβ) and hyperphosphorylated tau (p‐tau).^43^, ^44^ The abnormal accumulation of these toxic proteins leads to synaptic loss and neurodegeneration.^45^ Pathological tau seeding and spreading from the limbic to association cortices correlate more closely with cognitive impairment than Aβ.^46^, ^47^, ^48^ However, it is the synergistic interaction of both proteins^49^ and a third contributor, cerebral vessel disease,^50^, ^51^ that drives cognitive decline.^49^, ^52^ The typical pattern of these three pathologies is illustrated in Figure 1.

Nonetheless, overlapping pathologies and disease subtypes in mixed dementia cohorts do not align neatly with the classic description of AD, making mixed disease the most common presentation, whether in MCI or AD.^53^, ^54^, ^55^, ^56^, ^57^ We adhere to a biological definition of AD that emphasizes Aβ, tau, and neurodegeneration,^13^ while also acknowledging the contributions of vascular factors.^58^ Other comorbidities, such as neuroinflammation,^23^, ^59^ Lewy bodies,^60^ transactive response DNA binding protein 43 kDa (TDP‐43),^55^ and neuronal hyperactivity in the cortex and hippocampus,^61^ are discussed only briefly due to limited evidence from retinal studies on these pathologies.

## 4 *Post mortem* EVIDENCE

### 4.1 Brain

#### 4.1.1 Tau

Intracellular accumulation of hyperphosphorylated tau (p‐tau), forming neurofibrillary tangles (NFTs), occurs in subcortical structures about a decade before extracellular Aβ plaques appear in the cortex.^62^ P‐tau spreads through cell‐to‐cell transmission along neuronal pathways, particularly glutamate projection pathways in the neocortex.^63^, ^64^, ^65^, ^66^ Abnormal tau deposits appear to begin in the entorhinal cortex^64^, ^67^ and other medial temporal lobe areas,^68^, ^69^ gradually spreading to limbic structures along corticocortical pathways, ultimately affecting the entire neocortex (Figure 1).^62^, ^66^, ^70^ NFTs also appear in the spinal cord, even in Braak stage I,^71^, ^72^ though Aβ plaques are less common.^73^, ^74^

**RESEARCH IN CONTEXT**

1. **Systematic review**: The authors utilized an open‐ended search strategy to synthesize interdisciplinary knowledge on AD pathology and the potential of retinal imaging in estimating this pathology. Sources included evidence‐based resources, structured Medline searches using keywords from prior systematic reviews, citation tracking, and identification of key authors in the field.
2. **Interpretation**: Our findings suggest that while the utility of retinal thickness measured by OCT may be limited, choroidal thickness, retinal vessel imaging, and hyperspectral imaging show the most promising potential.
3. **Future directions**: The manuscript proposes increased focus on the aforementioned most promising retinal biomarkers. Examples of promising, yet less‐studied, retinal biomarkers include automatic subcellular segmentation of high‐resolution OCT images, as well as OCT optophysiology and elastography.

#### 4.1.2 Impact of tau on neurotransmitters

The spread of tau affects two modulation centers early in AD: one based on norepinephrine (noradrenergic) and another on acetylcholine (cholinergic). The cholinergic nucleus basalis of Meynert (nbM), part of the BFCS, is particularly vulnerable to tau^75^, ^76^ and modulates cognitive functions, including attention, memory, and arousal, through its connections to the neocortex and amygdala.^77^, ^78^ We will pay closer attention to the nbM later in this review.

#### 4.1.3 Aβ

Aβ is primarily produced by nerve cells that already contain p‐tau.^79^ Aβ plaques originate in the cerebral neocortex, spreading to the entorhinal cortex and eventually to subcortical structures,^80^ possibly through cell‐to‐cell transfer.^81^

#### 4.1.4 Cerebrovascular disease

Cerebrovascular disease, manifesting as small and large vessel disease, was found in 15% to 33% of a large autopsy series (*n* > 500) and 50% to 80% of cases with AD pathology.^54^, ^82^ This vascular pathology may account for 32% of the contribution of the aging process to dementia risk, while other factors, such as Lewy bodies and TDP‐43, may explain about 68%.^83^ AD‐associated cerebrovascular disease is characterized by increased Aβ deposits in cerebral arteries, resulting in cerebral amyloid angiopathy and disruption of the blood–brain barrier.^84^ This accumulation can then impair cerebrovascular reactivity and decrease perfusion, thereby accelerating the progression of AD.^85^, ^86^ In Braak stages III and IV, occipital angiopathy was more prevalent in those with cognitive impairment than in controls, while white matter arteriosclerosis showed no difference (*n* = 185).^52^

##### 4.1.4.1 Visual and circadian pathways

The visual cortex is relatively spared until the late stages of AD. Localized accumulations of AD pathology occur in the occipital neocortex during Braak stage 4, while significant involvement typically begins in stage 5 (Figure 1).^79^, ^87^, ^88^ The prevalence of cerebral amyloid angiopathy in the occipital lobe is notable, with rates of 60% to 80% in dementia cases^79^, ^89^, ^90^, ^91^ compared to 2% to 40% in non‐dementia cases aged over 65 years.^89^, ^92^, ^93^

Alzheimer's pathology eventually impacts subcortical structures within the visual and circadian pathways. The visual pathway comprises two components: the visual perception pathway and the ocular reflex pathway.

In the visual perception pathway, the lateral inferior pulvinar nucleus, lateral geniculate nucleus, superior and inferior colliculi, and primary visual cortex have been shown to exhibit both amyloid plaques and p‐tau NFTs.^94^, ^95^, ^96^, ^97^ The effect of Aβ plaques on the magnocellular^98^ or parvocellular layers^95^ of the lateral geniculate nucleus, a thalamic relay nucleus,^99^ remains inconclusive, although Aβ pathology appears more prominent than p‐tau in these structures.

**FIGURE 2:**
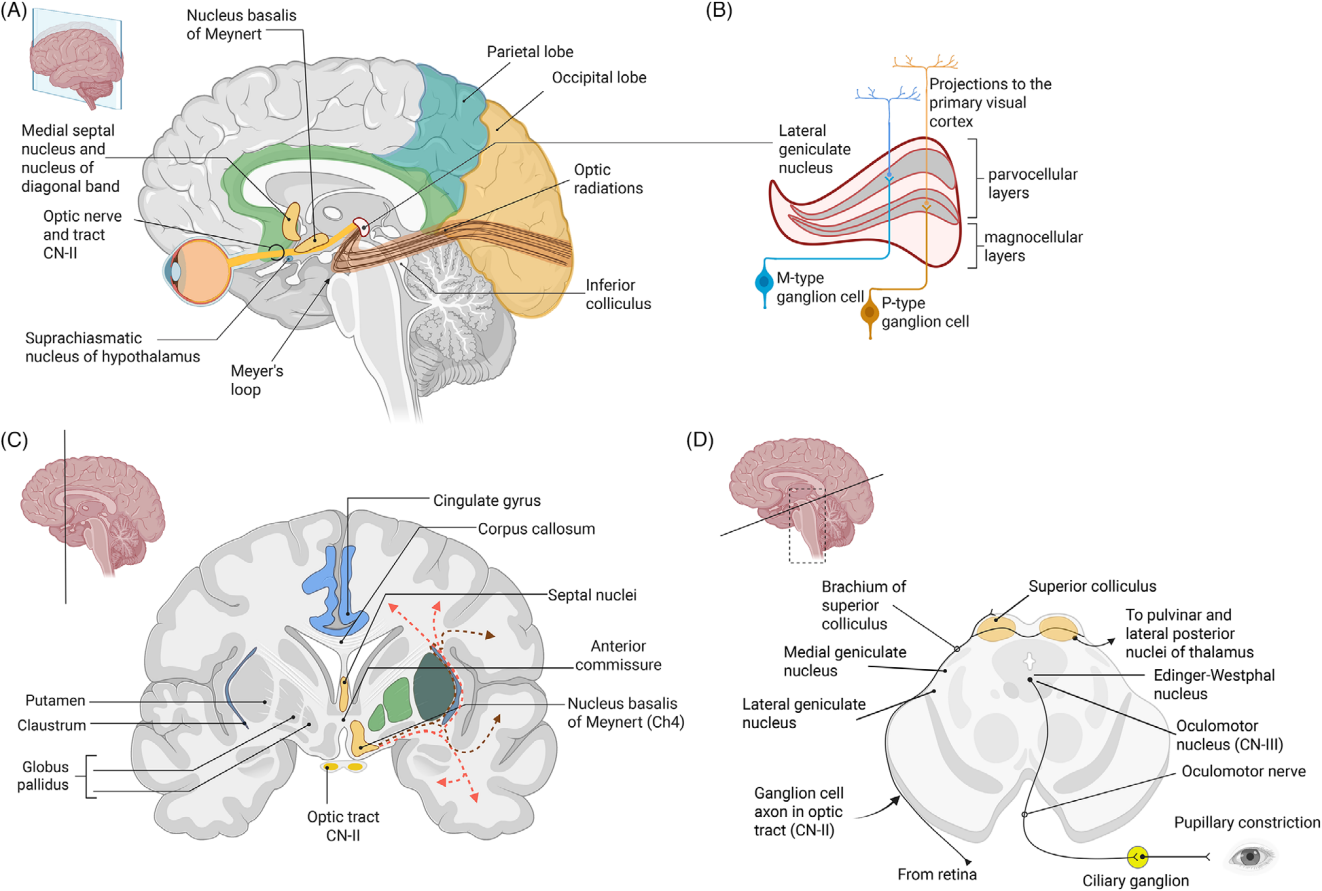
Brain anatomy. (A) Mid‐sagittal view of visual pathway shows that the visual cortex is relatively spared in early Alzheimer's disease (AD). Optic radiations traverse the temporal and parietal lobes. Projections from the lateral geniculate nucleus (LGN) relay visual information: Those from the superior visual field course rostrally into temporal white matter (Meyer's loop), then caudally to the visual cortex, while those for the macula and inferior field pass laterally around the ventricle and caudally through the parietal white matter. The hypothalamic suprachiasmatic nucleus, receiving inputs from melanopsin‐containing retinal ganglion cells, regulates sleep–wake cycles. One standard deviation variation in macular or pRNFL thickness correlates with approximately a 0.05 standard deviation variation in a brain volume, such as whole brain, frontal, parietal, temporal or occipital lobes, or hippocampus, when accounting for head size and age,^3^, ^4^ which appears too small for clinical usefulness. (B) Among over 18 classes of retinal ganglion cells, two major types are shown: M (magnocellular) cells, which detect motion and gross spatial features, and P (parvocellular) cells, which help identify color and form.^37^ Both LGN layers may be affected by amyloid plaques in AD. (C) A coronal cut at the nucleus basalis of Meynert (nbM) illustrates the basal forebrain cholinergic system, which supplies the hippocampus, olfactory bulb, amygdala, and neocortex,^362^ while the pontomesencephalotegmental complex of the brain stem innervates the dorsal thalamus and parts of the forebrain.^363^ The nbM has two projection pathways (medial and lateral) and is adjacent to the visual pathway, making it vulnerable to AD pathology and oxidative stress. The medial cholinergic pathway goes through the cingulum bundle and supplies several cortices, including the cingulate gyrus in blue. The lateral cholinergic pathway includes the capsular division in red (going through the external capsule and uncinate fasciculus) and the perisylvian division in brown (going through the claustrum and extreme capsule).^33^ (D) An axial midbrain cut shows the Edinger‐Westphal nucleus, which projects through CN‐III to the ciliary ganglion for pupillary constriction, and exhibits early AD pathology with exaggerated responses to acetylcholine antagonists.^100^ Figure created in Biorender.

In the ocular reflex pathway, the Edinger–Westphal nucleus, which receives retinal input, shows increasing neuronal loss with the progression of AD.^100^ This pathology is linked to exaggerated pupil dilation following cholinergic blocking agents, which may serve as a potential disease biomarker.^100^ In addition, the hypothalamic suprachiasmatic nucleus, part of the circadian pathway, shows accumulations of AD pathology (Figure 2).^101^, ^102^

### 4.2 Retina

#### 4.2.1 Retinal thinning

**FIGURE 3:**
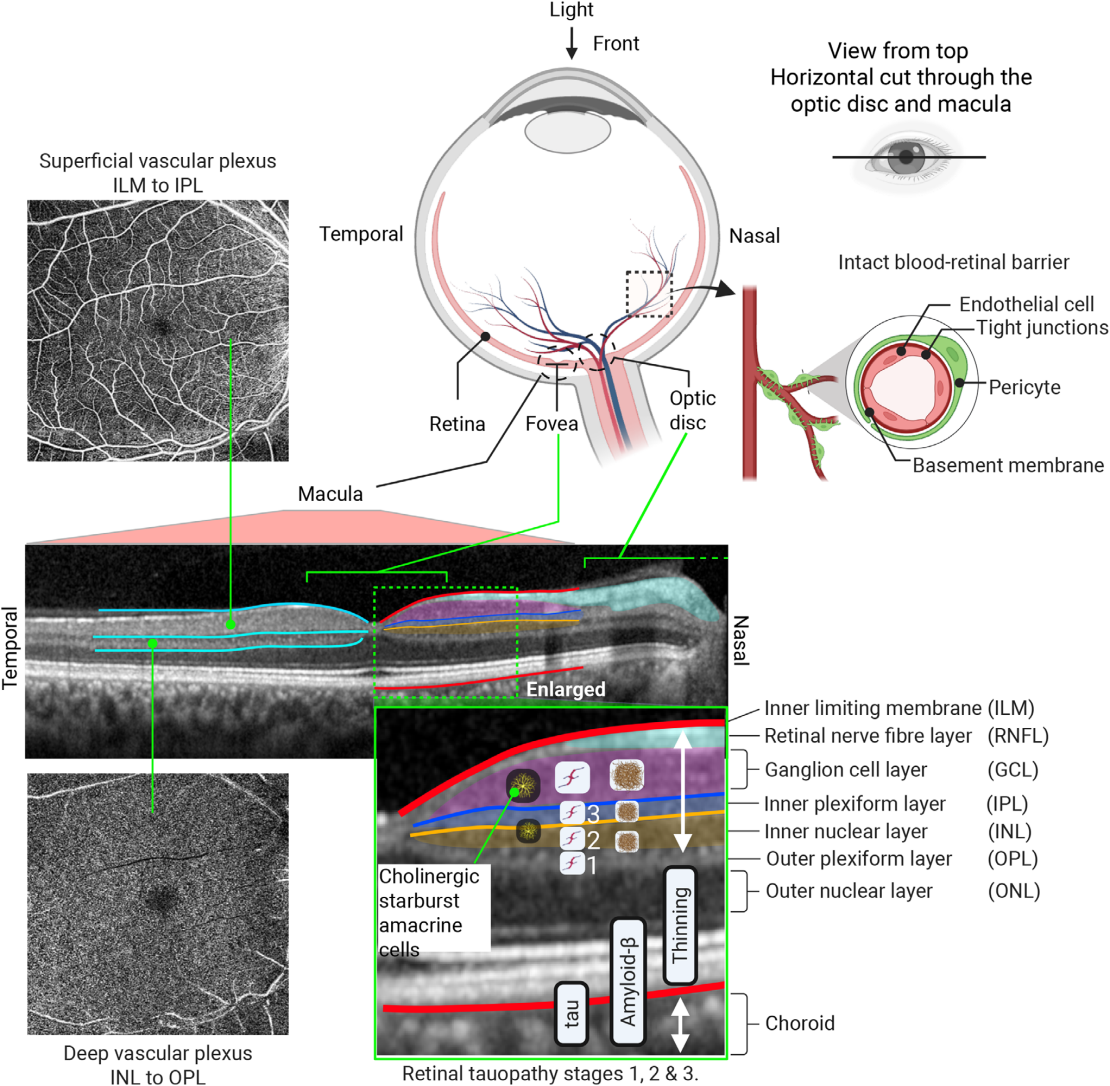
The retina and Alzheimer's disease (AD) pathology. Retinal optical coherence tomography (OCT) image of a 39‐year‐old male normal control is shown alongside patterns typical of AD pathology, including amyloid beta (Aβ) and tau deposits, and neurodegeneration with thinning in the inner retinal layers.^107^, ^118^ Note that tau can affect all layers.^25^ Retinal tauopathy appears distinct, progressing through staged involvement.^25^ The retinal nerve fiber layer comprises ganglion cell axons that form the optic nerve, reflecting the diencephalic origin of the retina. Starburst amacrine cells, primarily at the inner plexiform and inner nuclear layer border, with displaced ones in the ganglion cell layer,^141^ are key for acetylcholine neurotransmission and modulate direction‐selective ganglion cells.^139^ Their fate in AD, like that of brain cholinergic cells, remains understudied. Additionally, the choroid, a vascular tissue, shows reliably measured thinning in mild cognitive impairment compared to controls.^1^, ^24^ Figure created in Biorender.

Retinal thinning in AD was identified over a century after the first reports of brain atrophy in dementia.^103^, ^104^ *Post mortem* examinations show that the retina is thinner in patients with AD compared to controls, particularly in layers with acetylcholine‐producing cells, such as the ganglion cell layer (GCL), inner plexiform layer (IPL), and inner nuclear layer (INL) (Figure 3); however, data for MCI remain limited (Table 1). While retinal thinning may serve as a biomarker for neurodegeneration, it is unclear if retinal Aβ or tau deposits can differentiate AD from normal aging^105^ (Table 1; for animal studies, see Guo et al.^106^).

**TABLE 1:** Retinal *post mortem* examination in select studies.

	Thickness Alzheimer's disease versus controls		Amyloid beta Alzheimer's disease versus controls		Tau Alzheimer's disease versus controls	
Retinal layer	Difference	No difference	Difference	No difference	Difference	No difference
pRNFL	↓2,4,6			4,7,8		7,8
Total macula			9,10,13,14		14,1	
GCL	↓2,4,5,6		13	3,4,7,8,12	12	7,8
IPL	↓6			3,4,7,8,12	3,12,1^a^	7,8
INL	↓6			3,4,7,8,12	12,1^a^	7,8
OPL				4,7,8,12	3,1^a^	7,8
ONL	↓6			4,7,8,12		7,8,12
RPE				4,7,8,12		7,8,12
Choroid	↓11					

*Note*: ↓Denotes less thickness in Alzheimer's disease versus controls.

Abbreviations: GCL, ganglion cell layer; IPL, inner plexiform layer; INL, inner nuclear layer; OPL, outer plexiform layer; ONL, outer nuclear layer; pRNFL, peripapillary retinal nerve fiber layer; RPE, retinal pigment epithelium.

^a^ Layer‐by‐layer analysis is not provided in Hart de Ruyter et al.^97^ when differentiating cohorts, but most total tau and phosphorylated tau were observed in these layers. The retina is significantly thinner in Alzheimer's disease compared to healthy controls in *post mortem* studies, while differences in Aβ and tau between the two cohorts is less settled.

References: (1) Hart de Ruyter et al.,^97^; (2) Hinton et al.,^103^; (3) den Haan et al.,^107^; (4) Blanks et al.,^108^; (5) Blanks et al.,^109^; (6) Asanad et al.,^110^; (7) Ho et al.,^111^; (8) Williams et al.,^112^; (9) Lee et al.,^113^; (10) Koronyo‐Hamaoui et al.,^114^; (11) Tsai et al.,^115^; (12) Schon et al.,^116^; (13) La Morgia et al.,^117^; (14) Koronyo et al.^118^

#### 4.2.2 Aβ and tau in the retina

Data on retinal p‐tau and Aβ are limited. These proteins may help to identify AD, with increased retinal Aβ linked to greater cognitive impairment.^97^, ^107^, ^113^, ^114^, ^118^ Since p‐tau primarily differs between AD and normal controls in the far periphery of the retina,^97^, ^107^ detecting these proteins or their effects through retinal thinning may be beyond the field of view for most imaging methods. In a recent study from the Netherlands Brain Bank, the mean surface percentage of p‐tau positivity with Ser202/Thr205 (*b* = 23, SE = 8, *p* < 0.01) and Th217 (*b* = 10, SE = 5, *p* = 0.04) in the retinal far periphery could differentiate AD and Braak stages I to III NFT from normal controls with Braak stage 0.^97^ Both retinal p‐tau stains correlated with the Ser202/Thr205 stain in the hippocampus and temporal pole (b = 3 to 6, SE = 2, *p* < 0.01 to 0.02), with retinal p‐tau Ser202/Thr205 correlating further with its own variant in the medial frontal gyrus and parietal lobes. However, only 35% of AD (6/17) cases showed p‐tau in the retina.^97^

A recent *post mortem* study on MCI (normal *n* = 19, MCI *n* = 11, AD *n* = 24) found a difference in thickness between MCI and normal controls (15 to 20 µm) but not appreciably between MCI and AD (AD < MCI by ∼5 µm).^118^ This difference in thickness is larger than that reported in OCT studies (typically < 5 µm),^1^ likely due to the limited field of view in OCT imaging. Additionally, melanopsin retinal ganglion cells, which project to the hypothalamic suprachiasmatic nucleus to modulate circadian rhythm, show AD pathology.^117^

#### 4.2.3 *De novo* formation or cell‐to‐cell transfer

It remains unclear whether abnormal retinal Aβ and tau accumulation results from cell‐to‐cell transfer from brain cells, as seen in brain tau propagation^63^, ^119^, ^120^ or from *de novo* formation. Evidence supporting cell‐to‐cell transfer includes a non‐human primate model where injecting abnormal tau into the entorhinal cortex led to propagation to the visual system in the occipital lobe^121^; however, evidence of spread to the retina is lacking. In a non‐primate study, abnormal Aβ injected into one eye of a mouse propagated to the nervous system and the contralateral eye, resulting in retinal ganglion cell loss in both retinas.^122^

Conversely, in support of *de novo* formation, *post mortem* human studies indicate that a form of tau distinct from brain deposits accumulates in stages, starting from the outer plexiform layer (OPL) and progressing to inner layers such as the inner nuclear layer (INL) and IPL. These retinal deposit stages correlate with Braak amyloid phases but not tau stages or other brain pathology scores^25^; they appear smaller and rounder than the cored, diffuse, or fibrillary plaques in the brain^107^, ^111^ and do not show structures resembling fibrillar inclusions, threads, or NFTs.^97^ Surprisingly, in some mouse models, abnormal Aβ^114^ and tau^123^ deposits appear earlier in the retina than in the brain, supporting *de novo* formation.

#### 4.2.4 Correlation with Aβ and tau in the brain

Results on the correlation between brain and retinal pathology are mixed. Evidence shows abnormal Aβ^118^ and tau accumulation in both the brain and retina^107^; however, their morphology and biology may differ. In the brain, intracellular Aβ accumulation leads to cell death, release into the extracellular space, and plaque formation^124^; however, this may not occur in the retina.^113^ Retinal tau accumulation also leads to loss of retinal ganglion cells, and tauopathy‐laden ganglion cells increase in higher brain tau stages in MCI and AD (*n* = 41).^125^ One study, not accounting for age, found a strong unadjusted correlation (*r* ≈ 0.50 to 0.70, *n* = 21 to 32) between retinal thickness and abnormal Aβ deposits, tau staging, and Aβ phases in the brain (figure 1j,m, figure 5h, and figure S8h–j in Koronyo et al.^118^). For context, the hippocampal volume has a similar unadjusted correlation (*r* = −0.75, *n* = 16) with Braak NFT stages,^126^ which weakens (*r* = −0.23, *n* = 64) when considering age, sex, and time since death.^127^ While these findings are promising, *post mortem* studies on the retina are limited, with smaller sample sizes for each NFT stage (*n* = 12) compared to brain autopsy series.^107^

#### 4.2.5 Vessels, α‐synuclein/Lewy bodies, TDP‐43, and neuroinflammation

##### Vessels

Aβ deposits along retinal vessels disrupt tight junctions, mirroring findings in the brain, with markers of this disruption correlating with cerebral amyloid angiopathy in MCI and AD (unadjusted *r* = 0.67 to 0.75, *p* < 0.001, *n* = 38).^128^ Interestingly, fibro‐hyalinoid thickenings, resembling cerebrovascular disease changes, concentrate around optic disc vessels adjacent to peripapillary retinal nerve fiber layer (pRNFL) fibers that project through the visual pathway.^129^ Although most *post mortem* retinal vascular studies are not AD‐specific, evidence from hypertensive humans (*n* = 8) and primates shows vascular leakage linked to pericyte dysfunction,^130^ paralleling brain findings (see review by Kim and Cheon^131^). Calculated odds of observing cerebral artery sclerosis in the presence of retinal arterial sclerosis from older and larger pathology reports is somewhat weak (odds ratio [OR] = 1.9 [0.96 to 3.78], *p* = 0.064, *n* = 286).^132^, ^133^ Less examined are retinal veins, which appear to show signs of collateralization in the form of the vasa vasorum in aging.^134^ However, no studies were found in the context of AD or correlating these findings with cerebral veins.

##### α‐synuclein/Lewy bodies

α‐synuclein is rarely found in the retina in AD (1/19 [5%] of cases with brain Braak and Lewy body stage IV in the inferior retina) and is more likely present when the brain shows signs of α‐synucleinopathy, particularly in the brainstem, limbic, and neocortical areas.^135^ While α‐synuclein is primarily studied in Lewy body dementia and Parkinson's disease, AD can also exhibit this pathology.^51^, ^136^ In aging retinas, α‐synuclein and β‐synuclein are mainly in the IPL, while γ‐synuclein is typically in the nerve fiber layer. In AD, γ‐synuclein decreases in the nerve fiber layer but increases in the outer nuclear layer (ONL).^137^ Retinal α‐synuclein differs from that in the brain and does not correlate with brain Lewy bodies.^111^

##### TDP‐43

A small *post mortem* study (AD = 3, controls = 6), mostly focused on frontotemporal dementia, found no TDP‐43 deposits in either AD or control retinas.^138^

##### Neuroinflammation

Research on retinal neuroinflammation is limited. One *post mortem* study reported a two‐ to three‐fold increase in neuroinflammatory markers in MCI and AD retinas compared to normal aging, with strong but unadjusted correlations with retinal Aβ and tau deposits (*r* = 0.73 to 0.83, *p* < 0.01, *n* = 14 to 15).^118^

Vascular changes, proteinopathies, and neuroinflammation may interact to have significant implications for AD pathology in the retina; however, our understanding of their role is currently limited.

#### 4.2.6 Cholinergic cells

Acetylcholine modulates cognition in the brain and perception in the retina.^139^, ^140^, ^141^ Cholinergic cells in the retina have been identified through immunohistochemical staining for acetylcholine and choline acetyltransferase.^140^, ^142^ However, little is known about the fate of these retinal cholinergic‐producing cells (called starburst amacrine cells) within the aging–MCI–AD continuum (see Table S1 for a comparison of acetylcholine‐producing cells in the brain and retina). Retinal amacrine cells vary in type, with some producing dopamine that stimulates acetylcholine release from starburst amacrine cells. The interaction between dopamine and acetylcholine is reviewed by Witkovsky,^143^ while Faiq et al.^144^ discuss the role of acetylcholine in vision and its potential as a therapeutic target in retinal degeneration.

Although no *post mortem* examinations of the retina in preclinical AD have been reported, a recent study detected p‐tau, specifically p‐tau Ser202/Thr205 and Thr217, in amacrine and horizontal cells in the INL and the processes of amacrine cells in IPL in tauopathies, including AD samples.^97^ The impact of neurotransmitter loss in the brain on retinal tissues is still unclear. A study in rats showed that nerve growth factor applied to the conjunctiva enhanced cholinergic activity in the retina and forebrain,^145^, ^146^ potentially indicating a connection between the two acetylcholine‐producing tissues.

### 4.3 Limitations of animal models

While AD is primarily a human‐specific disease^147^ largely confined to the central nervous system,^30^, ^72^, ^148^ models of presumed causative agents, such as Aβ^149^ and tau,^121^ have been developed in non‐human primates.^150^ While these models show some related dysfunctions, such as gait abnormalities,^151^ they have not yet reproduced a comprehensive pathological, cognitive, and memory profile similar to human AD.^34^, ^147^ Identifying suitable animal models for studying the brain–retina association is challenging. For example, despite some non‐human primate models, such as the rhesus macaque and marmoset, developing signs of AD pathology in their aging brains, their retinas show minimal signs of aging.^152^, ^153^ Non‐primate mouse models have their own limitations, as discussed elsewhere.^154^, ^155^, ^156^

We now transition from *ex vivo* histopathological light microscopy, with a resolution of 0.2 µm, to in vivo imaging with significantly lower resolutions for retinal imaging (5 µm), brain MRI (0.5 to 3 mm), and brain positron emission tomography (PET) (2 to 8 mm).

## 5 MRI, PET, OCT imaging, and fluid biomarkers

### 5.1 Brain

#### 5.1.1 MRI: whole brain

##### Brain atrophy

Brain atrophy occurs in healthy aging and AD, but the overlap varies by region. Meta‐analytic “overlap percentages”^157^ reveal that aging and AD overlap 45% in whole‐brain volume, 34% in the temporal lobe, 25% in the hippocampus, 30% to 38% in the entorhinal cortex, and 51% in the basal forebrain. A 25% hippocampal overlap means only 75% of AD patients lie outside normal ranges.^157^, ^158^ Despite this overlap, specific patterns – cortical thinning in the medial and inferior temporal lobes, temporal poles, and regions of the frontal and parietal lobes – identify individuals at risk of cognitive decline.^159^, ^160^ Early AD shows atrophy in primitive allocortex regions, notably the hippocampus and entorhinal cortex.^161^, ^162^

A recent meta‐analysis reported hippocampal volume differentiates controls from AD (area under the receiver operator characteristic curve [AUC] = 0.91 [95% CI: 0.88 to 0.93]), but less accurately distinguishes controls from MCI (AUC = 0.73 [0.69 to 0.77]).^163^ In contrast, predicting MCI‐to‐AD decline is less accurate, with lesser sensitivity and specificity for the hippocampal volume (0.71 to 0.73 [0.64 to 0.80]) and for the medial temporal lobe (0.64 to 0.65 [0.51 to 0.76]) based on a meta‐analysis.^164^ Machine‐learning segmentation of hippocampus volume may improve diagnostic accuracy.^165^, ^166^

For the cholinergic system, posterior nbM volume yields the best diagnostic accuracy for differentiating normal controls from AD (AUC = 0.88 [0.84 to 0.92]),^167^ similar to the hippocampus. However, almost no studies on predictiing decline from MCI to AD based on BFCS volume could be found. The closest reported value is from one study predicting cognitive decline from BFCS volume (AUC = 0.78 [0.50 to 0.98]) in individuals with AD on cholinesterase inhibitor therapy.^168^ Interestingly, in preclinical AD (*n* = 147), the BFCS septal nuclei were found to be enlarged by 20 mm^3^ compared to controls (standardized mean difference [SMD] = 0.28),^169^ demonstrating that the earliest changes may not always involve atrophy.

In the later stages of AD, atrophy is evident in the whole brain and in the temporal, parietal, and frontal neocortices.^161^ Furthermore, spinal cord neuroimaging in AD is not well studied but may play a minor role in identifying the disease state.^170^ The number of studies on spinal cord imaging may increase with recent automated segmentation tools.^171^, ^172^

We will review the association of the aforementioned brain structures with retinal measures in Section 5.3.

##### Cerebral small vessel disease

Cerebral small vessel disease manifests as white matter hyperintensities on imaging^50^, ^173^, ^174^ and has an independent yet additive effect on Aβ deposition in AD pathogenesis.^51^, ^175^ Collagenosis of the large deep penetrating venular system in aging and dementia leads to vasogenic edema, capillary denudation, and oligodendroglia injury, contributing to cognitive impairment.^176^, ^177^ The degree of white matter hyperintensities within cholinergic pathways also correlates with impairment, particularly in executive function, attention, and memory.^178^, ^179^, ^180^, ^181^ Furthermore, remodeling of the carotid terminus secondary to hypertension affects the BFCS volume, correlating with greater cognitive impairment.^182^

White matter hyperintensities most frequently occur in the frontal and occipital lobes,^183^ which are associated with lower cortical thickness in the left superior parieto‐occipital cortex and lingual gyrus.^51^, ^184^ The gray matter volume of the lingual gyrus, part of the visual pathway, is associated with the pRNFL and GCL thickness,^5^, ^185^ with retinal thickness reflecting visual system changes. Given that the retinal blood supply comes from the anterior circulation, where hypertension has the most detrimental effects, the morphology of small vessels in the retina may provide insight into overall brain health in addition to retinal atrophy alone. As later described in Section 5.2.3, OCT imaging of retinal vessels helps distinguish healthy controls from MCI. In addition to vascular density loss, a compromised blood–brain barrier may lead to vascular edema and microhemorrhages, seen as amyloid‐related imaging abnormalities on brain MRI, during anti‐amyloid antibody therapy, occurring at rates of 1% to 40% depending on treatment regimen and apolipoprotein E ε4 carrier status.^186^, ^187^, ^188^

#### 5.1.2 MRI: visual and circadian pathways

##### Atrophy

While occipital lobe atrophy is not a recognized biomarker for identifying individuals at risk of cognitive decline or conversion to AD, its atrophy pattern can differentiate AD dementia with Lewy bodies from AD alone, with greater atrophy observed in AD.^189^ Given the connection between the retina and visual cortex, estimating occipital lobe atrophy using OCT may be feasible and provide insights into cognitive status in areas such as visuospatial skills and memory^190^; however, its clinical utility for diagnosing or predicting the prognosis of AD remains uncertain.

##### Cerebrovascular disease

Cerebral amyloid angiopathy is more prevalent in the occipital lobe, which is relatively spared in the early stages of AD. An age‐matched study found reduced white matter volume in the presence of cerebral amyloid angiopathy compared to controls or AD patients without cerebral amyloid angiopathy, particularly in posterior brain regions.^191^ In contrast, visual pathway white matter tracts are damaged in MCI and more so in AD, explaining visual defects such as impaired contrast sensitivity.^192^ Subcortical structures, including the pulvinar nucleus, lateral geniculate nucleus, superior colliculus, and suprachiasmatic nucleus, also show atrophy in AD.^101^ These changes occur earlier in the dorsal visual pathway than in the ventral pathway.^193^ Further studies on the visual pathway, including retinal imaging, are discussed in Section 5.3.

#### 5.1.3 Positron emission tomography

Neurofibrillary tau tangles identified in *post mortem* examinations can be detected in vivo using PET imaging. Imaging studies suggest that cognitive impairment may begin with isolated memory issues in Braak stages III and IV, with further progression beyond Braak stage III seemingly dependent on the presence of Aβ.^194^ Phases of Aβ cortical spread observed *post mortem* are similarly seen in vivo,^195^ correlating with transitions from preclinical AD to MCI to AD.^196^ PET findings indicate that these abnormal Aβ accumulations may begin 20 to 30 years before dementia onset.^197^ Combining fluorodeoxyglucose (18F) PET (FDG PET) with other imaging modalities and cognitive tests can enhance the sensitivity and specificity to >90% for predicting MCI to AD conversion.^198^ However, amyloid PET imaging is not widely available and is costly, with uncertain utility in MCI or cognitively unimpaired individuals.^199^, ^200^ Additionally, PET imaging positivity may vary by ethnicity.^201^ Despite these limitations, PET imaging influences management in approximately 60% of patients with MCI or dementia of uncertain origin, indicating its potential value.^202^ Its use is expected to rise with emerging disease‐modifying therapies.^203^

#### 5.1.4 Fluid biomarkers: cerebrospinal fluid and plasma

Nearly half of recent clinical trials on AD disease‐modifying therapy used a fluid biomarker (44%, 121/272 trials), with ∼25% using these biomarkers as target endpoints, mostly in phase 2 trials.^204^ These biomarkers include Aβ, tau, neurofilament light (neurodegeneration), and GFAP (astrogliosis/inflammation). A greater emphasis is placed on plasma biomarkers as they are less invasive compared to cerebrospinal biomarkers.

##### Cerebrospinal

A recent meta‐analysis supports the ability to identify individuals who will convert from MCI to AD based on cerebrospinal fluid Aβ1‐42, showing large effect sizes (SMD = 1.73 [1.39 to 2.07] for MCI converting to AD vs normal controls; SMD = 1.19 [0.96 to 1.42] for MCI converting to AD vs stable MCI) from 1092 participants across 22 studies.^205^

##### Plasma

Plasma p‐tau has emerged as a highly promising biomarker for AD detection. In particular, p‐tau217 demonstrates strong correlations with elevated PET Aβ (AUC = 0.92 to 0.96) and PET tau (AUC = 0.93 to 0.97).^206^, ^207^ Additionally, p‐tau181 effectively differentiates preclinical AD and MCI from controls (AUC = 0.82 to 0.90, *n* = 1298)^208^ and moderately predicts cognitive decline (AUC = 0.62 [0.53 to 0.71], *n* = 428).^209^ In contrast to tau, amyloid‐based plasma biomarkers, such as Aβ42/Aβ40, have a lower AUC (approximately 0.75) for distinguishing normal controls from AD,^210^ with similarly lower AUCs for identifying individuals with Aβ cerebrospinal fluid (AUC = 0.86)^211^ or brain PET Aβ (AUC = 0.75).^210^ However, they appear to be better suited than p‐tau181 for screening in anti‐amyloid therapy trials.^209^ Plasma neurofilament light chain (NfL) levels show genetic links to Aβ and tau production.^212^ They can predict cognitive decline, with a hazard ratio of 1.32 for progression from unimpaired or MCI to AD.^213^ However, NfL's ability to distinguish preclinical AD (AUC = 0.67) and MCI (AUC = 0.77) from controls is limited.^208^ While plasma biomarkers for Aβ and tau are not routinely used in clinical practice,^214^, ^215^, ^216^ ongoing advancements may improve their reliability^211^, ^217^, ^218^ and cost‐effectiveness for trial screening.^210^ Other plasma biomarker combinations may have similar diagnostic and prognostic value (*n* = 3366).^219^

Correlations with brain structural imaging further support the validity of plasma biomarkers. Plasma Aβ42/Aβ40 ratios correlate positively with hippocampal volume, whereas p‐tau levels inversely correlate with gray matter volume and cortical thickness.^220^ Additionally, plasma NfL levels correlate negatively with white matter microstructural integrity as assessed by diffusion tensor imaging.^220^

Overall, the AUCs for most biomarkers range from 0.70 to 0.90. Combining multiple fluid biomarkers has a minimal impact on accuracy.^208^

### 5.2 Retina

The retina undergoes thinning with aging, as shown by OCT imaging.^221^, ^222^, ^223^ The inner retinal layers get thinner while the outer layers thicken, necessitating separate assessments of each layer; the fovea also becomes thicker.^222^, ^223^ Despite these changes, both retinal layers and the pRNFL are typically thinner in MCI and AD compared to age‐matched healthy controls (Table 2). The in vivo thinning pattern in AD aligns with the accumulation of pathological proteins, primarily affecting the inner retinal layers that express acetylcholine, such as the GCL, IPL, and INL (Tables 2 and S1).^107^, ^110^, ^142^, ^224^ The following section provides an overview of select retinal imaging and electrophysiological biomarkers.

**TABLE 2:** Retinal optical coherence tomography examination in select meta‐analysis studies.

Retinal layer	Alzheimer's disease versus controls	Mild cognitive impairment versus controls	Preclinical disease versus controls
pRNFL thickness	↓1 to 5	↓3 to 5, 7, 8 (trending) 1, 6	=2
Inferior pRNFL	↓↓1 to 5	↓3, 4, 5, 8 / =2	
Superior pRNFL	↓↓↓1 to 5	↓2 to 5, 8 (trending)	
Nasal pRNFL	↓1 to 4 / =5	↓3, 4, 8 / =2, 5	
Temporal pRNFL	↓1 to 4 / =5	↓3 to 5, 8	
Macular volume	↓2 (trending)1	↓(trending)1, 2, 6	
Macular GC‐IPL thickness	↓1, 2	↓(trending)1 / = 2, 6	=2
Macular GCC thickness	↓1, 2	↓2, 6	
Foveal thickness	↓2	= 2, 7	
Outer layer thickness	↓2		
Subfoveal choroidal thickness	↓1, 2	↓2	

*Note*: ↓ denotes Alzheimer's disease (AD) < controls trending or statistically significant, = denotes almost equal.

Abbreviations: GCC, ganglion cell complex; GCL‐IPL, ganglion cell‐inner plexiform layer; pRNFL, peripapillary retinal nerve fiber layer.

There is greater retinal thinning in AD compared to healthy controls, which is less so in mild cognitive impairment or at the preclinical disease stage.

References: (1) Chan et al.,^1^ (2) Ge et al.,^24^ (3) Coppola et al.,^225^ (4) Thomson et al.,^226^ (5) Wang et al.,^227^ (6) Mejia‐Vergara et al.,^228^ (7) Noah et al.,^229^ (8) Knoll et al.^230^

#### 5.2.1 OCT resolution of 5 to 10 µm

##### Cross‐sectional studies

For retinal thickness to be adequate in screening, it must distinguish preclinical AD or MCI from normal controls. A recent meta‐analysis indicated that OCT parameters showed poor to moderate diagnostic accuracy with a low risk of bias, particularly in differentiating normal controls from MCI (GCL‐IPL: AUC = 0.55 to 0.72, mRNFL: 0.58 to 0.62, pRNFL: 0.57 to 0.64), with similar distinctions between controls and AD (AUC = 0.57 to 0.70)^2^ (Table 2).

##### Preclinical AD and MCI

Differences in pRNFL and inner macula measures (GCL and IPL) are negligible and below device resolution between normal controls and preclinical AD (SMD ≈ 0.05 [−0.5 to 0.5], *n* = 172 to 466), approximated from Figure 2 of Ge et al.^24^) or between cognitively normal twins with varying brain PET Aβ status (≤3 µm, *n* = 165).^231^ However, differentiation between MCI and normal controls shows slightly improved results (SMD ≈ 0.5 [−0.95 to 0.01]).^24^ Most studies involving MCI participants report significant pRNFL thinning, particularly in the amnestic MCI subtype^229^; however, these differences of 2 to 7 µm^1^, ^227^ often fall below the resolution of OCT devices, which is typically 5 to 7 µm. Choroidal thickness may be a more promising measure, with differences between MCI and normal controls appearing to be at least 10 µm for several sectors, exceeding the resolution of OCT devices (SMD of 0.5 µm for MCI compared to normal controls).^24^ However, no reports have compared choroidal thickness between preclinical AD and normal controls.

##### Alzheimer's disease

Studies consistently show pronounced thinning across all layers investigated, with the most significant thinning in the superior and inferior pRNFL quadrants. Meta‐analyses report that the total pRNFL is approximately 6 to 12 µm thinner in AD compared to controls, with the superior pRNFL being 8 to 24 µm thinner and the inferior pRNFL 7 to 21 µm thinner^1^, ^24^, ^225^, ^226^, ^227^; these values exceed the device's resolution. In contrast, the difference in inner retinal layers is only 3.7 µm [0.8 to 6.5], which is well below the resolution threshold.^1^ It is essential to note that even when mean differences exceed the device's resolution, the lower bound of the 95% CI may still be close to or below the resolution (<5 µm), effectively rendering these differences non‐significant. Choroidal thickness demonstrates the most significant differences, with a reduction of 65 µm [43 to 86], well above the device's resolution when comparing normal controls to AD.^1^

##### Longitudinal studies

Studies on retinal layer changes are limited. One study indicated that the rate of pRNFL or macular thickness change in just under 2 years between twins with (*n* = 16) or without (*n* = 129) cortical Aβ may not differ appreciably.^232^ However, in preclinical individuals at greater risk due to higher baseline brain amyloid levels, the IPL appears thicker,^233^ and the rate of IPL thinning is slower over 2 years.^11^, ^232^ Other studies present a mixed picture, with variable thicknesses in adjacent regions of each retinal layer when comparing MCI and AD.^234^, ^235^

##### Combining biomarkers for enhanced AD screening

Plasma and genome‐wide studies have shown AUCs of approximately 0.80 for distinguishing AD from controls (*n* = 1439)^236^ and 0.90 for identifying future conversion to AD from subjective memory decline or MCI when combined with other biomarkers (*n* = 883).^237^ While retinal imaging has a current diagnostic AUC 0.10 to 0.20 points lower than plasma biomarkers, it offers a low‐cost and rapid screening option. By combining the most promising retinal imaging techniques with other well‐performing biomarkers, diagnostic yields could potentially match those of established non‐invasive screening programs for other conditions, such as fecal immunochemical testing for colorectal cancer (pooled AUC = 0.87 [0.85 to 0.88])^238^ and mammography for breast cancer (pooled AUC = 0.89).^239^

#### 5.2.2 OCT resolution < 5 µm: adaptive optics and computational approaches

Current OCT devices have a resolution limited to 5 to 7 µm. A new OCT platform with a resolution of 3 µm and faster acquisition times allows for clearer imaging of cell contours and nuclei, including those of the GCL, ONL, and photoreceptor cells.^240^ Another approach combines adaptive optics with OCT. While the axial resolution of OCT is independent of pupil diameter, adaptive optics enhances resolution by compensating for lighting aberrations, achieving resolutions of 2 µm over a 300 × 300 µm field.^241^, ^242^ Most studies using higher resolution have focused on photoreceptors, particularly cone cells,^242^ with no research on MCI and AD at resolutions < 5 µm.

Greater sensitivity can also be achieved by increasing the number of scans and reducing the scan time.^243^ Similarly, higher acquisition rates in swept‐source OCT, combined with full‐field OCT, enable aberration correction through computational post‐processing, thereby improving visualization.^241^ A new wide‐field platform aims to provide an 8‐µm axial resolution and 3.1‐µm transverse resolution over a 3 × 3 mm area,^244^ which may enhance clinical utility.

Given that histological studies with resolutions < 1 µm and in vivo imaging at 5 µm have yielded mixed diagnostic results, the benefits of higher‐resolution in vivo retinal imaging for AD diagnosis remain uncertain. A higher resolution may allow a quantitative count of GCL cells, which may be a more effective diagnostic indicator than retinal thickness alone. Further reviews on this topic are provided by Ge et al., Hampson et al., and in chapters 9 and 10 of Drexler et al.^245^, ^246^, ^247^

#### 5.2.3 OCT angiography (OCTA), vascular reactivity, blood flow, and fundoscopy

##### OCTA: vessel density and fovea avascular zone

OCTA uses increased signal variance from moving erythrocytes to distinguish blood vessels from surrounding static tissue,^243^ quantifying the vessel density and the size of the fovea avascular zone, a foveal area devoid of blood vessels at the center of vision.

Cross‐sectional assessment: Recent systematic reviews present mixed findings on vessel density. Two studies on sporadic cerebral small vessel disease reported lower capillary density via OCTA (*n* = 137),^248^ while another found a negligible correlation between OCTA vessel density and white matter hyperintensity lesions on MRI (*n* = 30).^249^ Retinal vessel density, adjusted for age and sex, correlates strongly with cerebrovascular reactivity (partial *r* = 0.67) and weakly with cognitive functions linked to the frontal and occipital lobes, including executive and visuospatial functions (*β* = 0.02).^250^

The percentage area covered by vessels in the superficial and deep plexuses is 1% to 3% [0.02% to 6%] lower in MCI compared to controls, depending on the equipment used.^251^, ^252^ The fovea avascular zone is reported to be slightly larger in MCI compared to controls 0.07 [< 0.01 to 0.13] mm^2^, but no differences were observed between AD and controls, suggesting potential compensation for vessel loss in later stages or that differences in MCI were small to begin with given the small lower bound CI of differences (<0.01 mm^2^).^251^ The values mentioned are smaller than normative databases of healthy controls.^253^, ^254^ The lower bound 95% CIs of cohort differences for the fovea avascular zone exceed OCTA resolution, suggesting OCTA may be a reliable biomarker. However, differences in vessel density < 3% may be negligible given the resolution of devices and moderate to good repeatability (intraclass correlation: 0.65 to 0.85, *n* = 86).^255^ Newer segmentation methods may improve accuracy,^256^ but observed vessel density changes remain low (∼ 3%, *n* = 158 to 209),^256^, ^257^ especially in early disease. For recent reviews, see Yeh et al.^252^ and Xie et al.^256^

Longitudinal assessment: In a 2‐year study, MCI patients showed a 3% decline in superficial and deep capillary plexus density (*n* = 19), while age‐matched controls exhibited minimal change (*n* = 37, risk of conversion to AD high at 37%). The seven MCI patients that converted to AD displayed no further OCTA deterioration, likely because most vascular loss had already occurred.^258^ Such short‐term changes may be negligible given the device resolution and only moderate to good measurement repeatability.^255^

##### Vascular reactivity and blood flow

The Dynamic Vessel Analyzer employs OCT imaging with light flicker to induce vasodilation followed by vasoconstriction, a biomarker of vascular reactivity. It also uses multispectral imaging to assess blood oxygenation. A significant arteriovenous oxygenation difference is noted when comparing MCI/AD to controls (SMD = 0.70).^259^ Limited data from two studies on light flicker‐induced vasodilation show mixed results, with negligible differences in arterial dilation between MCI/AD and controls (SMD = 0.10, *n* = 90)^259^ and medium‐sized differences when vascular risk factors were greater in AD (MCI vs controls: SMD = 0.4; AD vs controls: SMD = 1.6, *n* = 56).^260^ Vascular risk factors in the control group were not reported. Larger differences were observed when measuring the difference between flicker‐induced arterial dilation and post‐flicker vasoconstriction (MCI vs controls: SMD ≥1, *n* = 12 MCI, *n* = 32 controls; AD vs controls: SMD ≥ 2, *n* = 12 AD).^260^

The Retinal Function Imager provides in vivo retinal perfusion maps and blood flow rates, showing large differences in blood flow relative to retinal capillary density when comparing AD to controls (SMD = 2, *n* = 34, accounting for vascular risk factors).^261^ Similar findings are noted for choroidal blood flow based on OCTA (SMD = 0.85, *n* = 36, age‐ and sex‐matched).^262^ We did not find data on MCI or preclinical states.

While the aforementioned studies focused on middle‐aged and older adults, arteriolar and venular diameters from fundus photography can estimate brain structural measures in adolescents with or without bipolar disorder,^263^ which may be applicable to early AD detection in younger populations. Other retinal vascular changes, such as retinal hemorrhages in individuals with cerebral amyloid angiopathy, may also serve as specific biomarkers or indicate hypertensive or sporadic cerebrovascular disease.^248^

##### Retinal fundus photography

Retinopathy of any kind on fundoscopic images, such as focal arterial narrowing, has a small association with dementia (OR = 1.51 [1.14 to 1.99]) or MCI (OR = 1.97 [1.27 to 3.06]).^264^ Retinopathy of any kind similarly correlates with white matter hyperintensities and lacunar or cerebral infarction (OR = 1.94 to 1.99; 95% CI: 1.21 to 3.25; *n* = 3092 to 11,099), even after adjusting for vascular risks and study quality.^265^ However, the longitudinal association with cognition fades, whereas associations with neuroimaging markers of atrophy or stroke persist.^264^

Overall, early studies suggest medium to large effects for eye vascular studies, indicating that retinal and choroidal vascular imaging may be more promising than retinal thickness, providing valuable insights beyond traditional vascular risk factors.

#### 5.2.4 OCT and electroretinography

Electroretinography measures retinal electrical activity in response to light, revealing GCL dysfunction in preclinical AD in two studies (*n* < 50).^266^, ^267^ Pattern electroretinograms show similar performance to hyperspectral imaging (AUC = 0.84 [0.72 to 0.95], *n* = 29)^266^ using photopic negative response amplitude and N95 implicit time^266^ as estimators of GCL dysfunction.^268^ Preclinical AD participants with higher Aβ loads can be identified with good accuracy using electroretinogram and GCL‐IPL thickness (AUC = 0.90 [0.81 to 0.99], *n* = 49).^267^ However, as a limitation, performing an electroretinogram can be cumbersome.

#### 5.2.5 OCT optophysiology and elastography

##### Optophysiology and neuronal hyperexcitability

Increased neuronal activation in the cortex and hippocampus is linked to faster cognitive decline, followed by hypoactivation in later disease stages.^61^, ^269^ One non‐invasive method for estimating neuronal excitability in the retina is to measure the intrinsic optical signal from photoreceptors stimulated by light. In a study using the 3xTg‐AD mouse model, the intrinsic optical signal showed a significant difference compared to controls by month 4 (0.29 ± 0.04 vs 0.23 ± 0.06, SMD = 1.2, *n* = 22, figure 4B2 of Ma 2023^270^). This neuronal hyperactivity signal appeared 1 month earlier than structural changes, such as inner retinal thickness loss, which became evident by 6 months. This inner retinal thickness loss can be observed in humans with MCI aged 50 to 60 years.^1^

##### Elastography

Future clinical applications of OCT elastography may enable the detection of arteriosclerosis in the choroid or assessment of the elasticity of other retinal layers.^271^, ^272^ However, this may primarily apply to the middle and outer choroidal layers, which contain medium‐ to large‐sized vessels, as the inner layers consist of fenestrated capillaries.^273^ Research on retinal tissue elasticity changes in AD remains limited, and studies of brain magnetic resonance elastography in AD are in the early stages.^274^

Currently, no human studies have examined retinal neuronal hyperactivity or OCT elastography.

#### 5.2.6 Imaging retinal amyloid: fluorescence and hyperspectral imaging

Detecting Aβ in the retina is challenging, as only 0.6% of the retinal surface contains these deposits.^275^ *Ex vivo* and in vivo studies have attempted to use curcumin for autofluorescence imaging of retinal Aβ^276^; however, the results remain uncertain.^277^ A review of six studies involving >100 participants highlighted that most Aβ is located in the far periphery of the retina, complicating detection due to overlap with other conditions, including age‐related macular degeneration and aging. Additionally, automatic quantification methods are lacking, and confounding factors, such as age, sex, vascular risk factors, or head size, were often not included in analyses correlating retinal findings with brain or fluid biomarkers.^276^ Two methods that show the most promise are discussed next.

##### Fluorescence lifetime imaging ophthalmoscopy (FLIO)

This method measures the duration of natural autofluorescence in the retina. A pilot study (*n* = 15) indicated a small unadjusted correlation between FLIO parameters and cerebrospinal Aβ (*r* = −0.5 to −0.6) and tau (*r* = 0.5 to 0.6) levels. However, age differences between groups may have influenced these results.^278^ FLIO lifetimes increase with age (*r *= 0.60 to 0.80, *n* = 97),^279^ as do cerebrospinal Aβ concentrations.^280^

##### Hyperspectral imaging

This method captures images across a wide range of wavelengths to identify distinct patterns associated with Aβ. In a *post mortem* animal study, this technology successfully detected Aβ in the neocortex and vessel walls of aged squirrel monkeys.^281^ In vivo human retinal imaging can detect brain Aβ based on PET, with the presumption that hyperspectral imaging can detect retinal amyloid deposition in early AD (AUC = 0.82 [0.67 to 0.97] [*n* = 35]) with good accuracy. Hyperspectral scores correlate with PET Aβ Centiloid values based on a small study (*r* = 0.46 [0.13 to 0.69], *p* = 0.008, *n* = 33), but with a wide range of plausible values.^282^ Other studies on hyperspectral imaging combined with additional retinal biomarkers report similar AUC values of 0.74 to 0.85.^283^, ^284^ Research on imaging tau is ongoing, potentially using a label‐free spectral signature of p‐tau.^285^ For a review on Aβ, tau, and hyperspectral imaging in the retina, see Tang et al.^276^

##### Exploring additional imaging modalities

This review focuses on retinal structural imaging using OCT and only briefly mentions fundus photography. While most studies have emphasized structural imaging, evidence on in vivo imaging of Aβ in the retina and its association with brain Aβ accumulation,^286^ total gray matter,^287^ hippocampal volume,^288^ and global cognition^288^ is accumulating. Notable examples include a proof‐of‐concept study that detected Aβ deposits in fundus imaging correlating with PET levels (*n* = 4)^289^ and findings that retinal vessel pulsation in response to flicker‐induced light stimulation positively correlates with brain Aβ in PET.^290^ Currently, there is no in vivo retinal imaging of tau in humans. For a review of other ocular biomarkers not discussed here, refer to Wu et al.^291^

### 5.3 Brain–retina correlation: brain imaging, fluid biomarkers, and retinal imaging

#### 5.3.1 Magnetic resonance imaging

Significant associations between retinal OCT and brain structural MRI are mixed, primarily involving pRNFL and inner retinal layers, especially the GCL, with gray and white matter in the temporal, parietal, and occipital lobes, as well as the hippocampus, while frontal lobe associations are less common (Table 3, Table S2). The temporal lobe and the hippocampus are important for diagnosing AD and predicting cognitive decline.^292^

**TABLE 3:** Significant associations in brain MRI and retinal optical coherence tomography in select studies.

							GM+WM	
Retina	Frontal	Temporal	Parietal	Occipital	BG‐Th	Total	HP	Total
**Gray matter (GM)**								
Macula								
GCC	1			1		1	1	1
GCL		2, 7^a^	2	3, 2	3	2	2	
IPL		2	2	2		2		
GC‐IPL		8, 11		1, 8		1	12	1, 12
Fovea				11				
mRNFL					4	1	1, 4	1, 4
mTotal	1	7, 10	4, 9, 10	1, 4	4	1, 9, 10	1, 4	1, 4
pRNFL	2	2, 5^a^, 7^a^, 8		3, 2, 5^a^, 11	3	2	2, 6, 5^a^	
**White matter (WM)**								
Macula								
GCC						1		
GCL	2	2, 7^a^	2	2		2		
IPL		2	2	2		2		
GC‐IPL						1		
Fovea								
mRNFL					4			
mTotal		7^a^	4	4	4			
pRNFL	2	2, 5^a^, 7^a^		2	12	2	12	12

*Note*: Refer to Table S2a for more details on each included study in the footnotes. Refer to Table S2b for non‐significant effects. Refer to Table 4 for effect sizes of whole brain and temporal lobe structures.

Abbreviations: BG‐Th, basal ganglia / thalamus (central or subcortical) brain region; GCC, ganglion cell complex; GCL, ganglion cell layer; GC‐IPL, ganglion cell‐inner plexiform layers; GM, gray matter; HP, hippocampus; IPL, Inner plexiform layer; mRNFL, macular retinal nerve fiber layer; pRNFL, peripapillary retinal nerve fiber layer; WM, white matter.

^a^ Indicates that grey and white matter were reported together and not separately.

References: (1) Chua et al.,^3^ *n* = 2131; (2) Mutlu et al.,^4^ *n* = 2124; (3) Mutlu et al.,^5^ *n* = 2235; (4) Sergott et al.,^6^ *n* varied by brain region = 656 to 1111; (5) Shi et al.,^185^ *n* = 80; (6) Mendez‐Gomez et al.,^293^ *n* = 97; (7) Casaletto et al.,^294^ *n* = 79; (8) Ong et al.,^295^ *n* = 164; (9) den Haan et al.,^296^ *n* = 30; (10) den Haan et al.,^297^ *n* = 134; (11) Mejia‐Vergara et al.,^298^ *n* = 20; (12) Barrett‐Young et al.,^299^ *n* = 818 to 828.

References with *n* > 100 and adjusted for age and sex: (1) Chua et al.,^3^ (2) Multu et al.,^4^ (3) Multu et al.,^5^ (4) Sergott et al.,^6^ (8) Ong et al.^295^

The Rotterdam studies^4^, ^5^ suggest that changes in the pRNFL and GCL may be associated with brain white matter changes that are not visible on structural imaging but can be detected via diffusion tensor imaging. The GCL was better at estimating visual pathway structures, while the pRNFL was better at estimating global brain changes.^5^ A selection of these white matter regions, such as the corpus callosum, connect visual cortices but are not traditionally part of the visual pathway.^300^, ^301^

Research can be furthered by studying the association between retinal measures, such as pRNFL thickness, and white matter pathways responsible for cognitive modulation that are vulnerable to AD pathology, including the cholinergic projection pathways from the nbM to the neocortex.^179^, ^302^, ^303^ While limited research exists on using OCTA to estimate white matter tract damage in the brain, current evidence shows that superficial plexus OCTA measures have negligible correlation with white matter hyperintensities on MRI in normal controls or those with cognitive decline (*β* = −0.04 to 0.05).^304^

Effect sizes vary; correlations between the whole brain or hippocampus with retinal thickness measures are generally small (*r* = 0.12 to 0.23, *n* = 948 to 1111)^6^ but can be medium to large in smaller studies (*r* = −0.44, *n* = 30)^296^ (Table 4, Table S3), with the magnitude of the correlation halving when adjusting for head size.^299^ Standardized regression coefficients considering head size, age, and sex are typically small (*β* = −0.02 to −0.10) for the whole brain and its smaller structures, such as the hippocampus.^3^, ^4^

**TABLE 4:** Effect size magnitude and direction when estimating whole brain or temporal lobe structures from retinal thickness in select cross‐sectional studies.

Reference	Whole brain	Temporal lobe / HP	Coefficient type	Adjusted for	Analysis sample
Chua et al.^3^	−0.08	−0.03 to −0.05	Standardized regression coefficient	A, H, S, o	2131
Multu et al.^5^	Positive association^a^	Positive association^a^	^a^	A, H, S, o	2235
Mutlu et al.^4^	−0.03 to −0.05	−0.03 to −0.06	Standardized regression coefficient	A, H, S, o	2124
Sergott et al.^6^	0.23	0.12	Pearson *r*	‐	948 to 1111
Mendez‐Gomez et al.^293^	0.17^b^	0.01^b^	Unstandardized regression coefficient^b^	A, S, o	90 to 97
Shi et al.^185^	‐	0.12 to 0.06	Pearson *r*	‐	80
Casaletto et al.^294^	‐	0.23 to 0.30^c^	Standardized regression coefficient	A, H, S, o	75
Ong et al.^295^	‐	−2.32 to −2.77^d^	Standardized regression coefficient	A, H, S, o	164
den Haan et al.^296^	−0.44	‐	Spearman *r*	‐	30
den Haan et al.^297^	< 0.01 to −0.31	−0.11 to −0.20	Standardized regression coefficient	A, S	134
Mejia‐Vergara et al.^298^	‐	0.03 to 0.04	Standardized regression coefficient	A, o	20
Barrett‐Young et al.^299^,^e^	0.04 to 0.14 −0.08 to −0.09	0.02 to 0.10	Standardized regression coefficient	H, S, o	818 to 828

*Note*: See Table S3 for details. Retina thickness is measured by optical coherence tomography. Effect sizes are based on standardized regression or correlation coefficients. These effect sizes are typically smaller (by 10 folds at times) when accounting for head size and age for brain volumes and just age for cortical thickness in comparison to unadjusted regression coefficients or Pearson's r. The direction of effect is not always the same in different studies, even when using similar predictors and outcomes, possibly indicating that effect sizes are close to zero (no effect).

Abbreviations: A, age; H, head size in model as predictor or fraction of brain volume to head size used; HP, hippocampus; o, other variables such as vascular risk factors; S, sex.

^a^ Based on result description and positive t‐values of the voxel‐based morphometry analysis output; no coefficients provided for regression models based on brain volumes.

^b^ Per 10 μ m retina change, which is close to one standard deviation change in retinal measure.

^c^ An outlier study in that effect sizes are large despite accounting for age and head size.

^d^ Magnitude not comparable to other studies as retinal measure is outcome not predictor.

^e^ Study could not assess impact of age, as all ages were 45.

Placing these effects sizes in a clinical context is particularly relevant when considering the typical macular thickness difference in the population (39 µm) or between normal controls and AD (5 to 15 µm),^1^ as well as the typical differences in hippocampal (1 to 2 cc) and total gray matter (10 to 25 cc) volumes between normal controls, MCI, and AD.^305^ For example, a 1 standard deviation change in total macular thickness (30 µm) corresponds to a minimal estimated change in hippocampus volume (0.02 cc) or total gray matter volume (2.4 cc), which may lack clinical utility (values are from UK Biobank data fields: 27800, 27801, 26641, 26663, and 25005, the difference in population [39 µm] is in the first and ninth deciles in the UK Biobank, and regression estimates are from Chua et al.^3^).

When examining the total retina versus retinal layers, the occipital lobe shows above‐average associations with retinal measures, but they remain small (−0.05 to −0.08). Standardized regression coefficients show little change (0.01 to 0.04) when comparing total macular thickness to inner retinal layers,^3^, ^4^ indicating limited gains in measuring individual retinal layer thickness (Tables 2 and 4).

Limitations in our discussion include the lack of a formal meta‐analysis to separate diagnostic group effects and the limited number of longitudinal studies,^306^, ^307^ which show minimal changes (<5 µm) in retinal layer thickness over a 2‐year follow‐up period.^11^, ^232^ Longitudinal cohorts, such as the UK Biobank^3^ and Dunedin study,^299^ may provide future insights with longer follow‐ups.

#### 5.3.2 Positron emission tomography

Data on the association between brain Aβ or tau on PET and retinal imaging are limited. A small to medium negative correlation exists between changes in macula thickness and the PET amyloid standardized uptake value ratio in a 1‐ to 2‐year follow‐up of a small sample (*r *= −0.09 to −0.20, *n* = 11 to 12) when not accounting for confounders. Assuming this correlation is equivalent to an unadjusted *β* in a regression model,^308^ a 10‐µm decrease in retinal thickness corresponds to a 0.01 increase in the PET amyloid uptake value.^6^ Given typical PET uptake values (0.5 to 3)^309^ and previously mentioned retinal thickness variations between cohorts or in the population (5 to 39 µm), this results in a negligible change of 0.05 in PET values detected by retinal thickness measures.

Upon closer examination of individual retinal layers, the IPL, which appeared thicker at baseline in one study, underwent slow thinning during a 2‐year follow‐up in preclinical AD individuals with brain Aβ deposits.^11^, ^232^, ^233^ The initial association of IPL with brain Aβ in that study in which IPL appeared thicker at baseline (SMD = 0.88, *r* = 0.31, *p* = 0.029, *n* = 63)^233^ was lost in the longitudinal follow‐up (b = −0.014, *p* = 0.144, *n* = 56).^11^ Instead, the macular RNFL thickness change was negligibly associated with the brain Aβ change over 2 years (*r* = 0.02) when accounting for age.^11^ The authors^233^ noted retinal inclusion bodies in the IPL, potentially containing Aβ, and highlighted similarities between the IPL and neocortex, both showing changes in regions with high cholinergic synaptic activity and Aβ accumulation. This initial increase in IPL thickness is similar to the findings in the earlier study mentioned in Section 5.1.1, regarding cholinergic septal nuclei in cognitively normal individuals at risk for AD, which also demonstrated increased volume.^169^ This suggests that early signs in preclinical AD may not always involve atrophy.

At least one study found no association between retinal GCL thickness and Aβ PET,^290^ indicating mixed results regarding retinal thickness measures and brain Aβ. However, that study did show an association between Aβ PET and retinal and venular artery pulsation.^290^ A validation of these associations will depend on ongoing studies.^310^, ^311^, ^312^, ^313^, ^314^

While data are limited on the correlation of brain Aβ PET with retinal amyloid imaging or an electroretinogram, hyperspectral retinal imaging (*r* = 0.46 [0.13 to 0.69]),^282^ curcumin‐labeled autofluorescence deposit counts (*r* = 0.65 [−0.09 to 0.9]),^289^ and a composite measure of visual event‐related potentials (*r* = −0.54 [−0.70 to −0.30])^315^ suggest small to medium unadjusted correlations (values derived from figure 4a of Javitt et al.,^315^ with the 95% CIs calculated based on the methodology outlined by Cohen et al.^316^). These effect sizes may be further influenced by age and other confounders.

#### 5.3.3 Fluid biomarkers: cerebrospinal fluid and plasma

##### Cerebrospinal fluid

The association of cerebrospinal Aβ or tau with pRNFL or perifoveal retinal thickness is small when accounting for age and sex (*β* = 0.01 to 0.14 in one study [*n* = 90],^297^ not significant in an earlier study by the same authors [*n* = 30],^296^ and *β* = −0.07 to 0.05 in another [*n* = 99]^317^) (the last study's^317^ coefficient was recalculated based on a 12‐µm standard deviation for average pRNFL^308^, ^318^). Stronger associations were noted without considering confounders (*r* = −0.3 to 0.4).^319^

##### Plasma

The association of plasma p‐tau181 and NfL with pRNFL or GCL‐IPL thickness (*β* = −0.06 to −0.18, *n* = 431 to 500) when not considering age appears weak.^320^ The same is true with p‐tau181, p‐tau217, Aβ42/40, NfL, and more novel indicators of retinal gliosis when considering age (recalculated *β* from Figure 1 estimated at <0.05, *n* = 82).^321^

No studies on Aβ or GFAP plasma biomarkers were found.

## 6 Study design and statistical considerations

### 6.1 Diagnostic criteria

For studies assessing diagnostic accuracy, aligning the diagnosis with the current biological definition of AD is preferred^13^; however, the biological definition may not always lead to clinical progression to dementia.^200^ A meta‐analysis of retinal imaging studies using the biological definition of AD indicated a negligible to small effect size in differentiating controls from AD (SMD = −0.56 [−0.92 to −0.21] for macular RNFL and −0.54 [−1.22 to 0.13] for pRNLF thickness),^322^ suggesting uncertainty in its utility. If biological definitions are costly or inaccessible, established country‐ or society‐specific guidelines for AD^323^, ^324^ or MCI^325^ diagnosis can be used. In screening studies, there may be less emphasis on exact diagnoses when assessing general correlations, such as with hippocampal volume.

### 6.2 Confounders: *post mortem* time interval, head size, and axial length

The time from death to tissue preservation impacts gene expression and brain subcellular structures^326^; thus, it is essential to include this interval as a confounder in the analysis. Evidence for retinal changes *post mortem* is limited and not quantified in terms of macular thickness changes.^327^ OCT images suggest retinal detachment may occur within 30 min^247^ and retinal edema within the first 6 h *post mortem*.^328^ However, overall thickness changes are minimal after 48 h,^329^ likely due to shrinkage or deformity alongside edema and detachment.

Age and head size must be considered when assessing brain volume, while cortical thickness assessments should account for age, sex, and scanner characteristics.^330^ Although MRI‐derived intracranial volume is unavailable at screening without neuroimaging, head circumference can serve as a surrogate, moderately correlating with MRI‐derived intracranial volume (*r* = 0.65 to 0.70, *n* = 99).^331^ No studies have measured the correlation between eye axial length and intracranial volume; however, axial length moderately correlates with head circumference (*r* = 0.60, *n* = 40),^332^ suggesting a smaller correlation with intracranial volume (*r* ≈ 0.40).

Incorporating eye axial length into modeling may be beneficial, particularly for measures involving the transverse plane, such as macular volume.^333^ If unavailable from optical biometry, axial length can be estimated from brain MRI with an accuracy of 0.1 to 0.8 mm compared to the reference standard, which is clinically acceptable.^334^ When including axial length alongside head size, it is best to check for multicollinearity to ensure the reliability of regression coefficients.

### 6.3 Side and multilevel modeling

For right and left retinal measures, several approaches exist: (1) using one side predominantly,^5^ (2) randomly selecting either side,^3^ (3) using the lowest value from either side,^293^ (4) reporting both sides separately,^6^ (5) averaging values,^297^ or (6) entering side as a random effect in a multilevel model. An exploratory analysis can help determine the best approach based on side differences and associations with outcomes.

### 6.4 Multiple testing, effect size, uncertainty of estimates, and clinical usefulness

Given the numerous sectors in retinal imaging studies, conducting factor analysis or dimensionality reduction may simplify the analysis by aggregating sectors through averages or latent components. This approach can help identify clinically relevant effects in smaller samples (*n* < 100) by reducing the number of multiple tests.

Effect size cut‐off suggestions for gerontology research indicate that Pearson *r* values of 0.10, 0.20, and 0.30 are small, medium, and large, respectively, while between‐group SMDs (Cohen's *d*) or Hedges’ *g* values of 0.15, 0.40, and 0.75 are similarly categorized.^335^ Pearson or Spearman correlation coefficients can be used to calculate standardized regression coefficients in simple linear regression,^308^ and the aforementioned cut‐off values may also be used to interpret the magnitude of standardized regression coefficients.

The *f* ^2^ effect size can compare the variance explained by models with and without retinal biomarkers, quantifying the retinal contribution toward the estimation^336^, ^337^, ^338^:

$$f^{2}=\frac{R_{fullmodel}^{2}-R_{reducedmodel}^{2}}{1-R_{fullmodel}^{2}}$$

Here, *R* ^2^ represents the variability in the outcome measure, such as hippocampal volume, that is explained by the model using predictors including retinal measures and age. Both models include all non‐retinal variables, differing only in the inclusion of retinal measures in the full model. *f* ^2^ values of 0.02, 0.15, and 0.35 are considered small, medium, and large effects, respectively.^337^, ^338^

Interpreting effect sizes is most meaningful after considering confounders through partial correlation or multiple regression coefficients. Methods also exist for quantifying a predictor's influence on an outcome by calculating a variable importance score.^339^ For instance, a score >0.8 may indicate a useful predictor in models such as partial least‐squares regression.^340^

Most studies report regression or correlation coefficients and *p* values. Reporting standardized regression coefficients alongside measures of uncertainty of the estimate, such as standard error or CIs, aids in comparing effect sizes across models and studies.^308^ It is important to consider the lower bound of the CI to ensure that the smallest plausible differences exceed the resolution of the imaging device (>5 µm) or are clinically meaningful; otherwise, the results may lack clinical utility.

In summary, accounting for readily available confounders, such as age and sex, ensures that retinal OCT adds meaningful information. Measures such as head circumference or MRI‐derived intracranial volumes should be included in models estimating brain volumes. In *post mortem* studies, results should be adjusted for the time from death to data acquisition. Recommendations for harmonizing methodologies were summarized previously by Alber et al.^27^

### 6.5 Other considerations and study limitations

In several investigations, retinal measures were treated as outcomes (dependent variables),^9^, ^295^, ^321^, ^341^ a distinction that can affect study interpretation and pooling of effect sizes.

The repeatability of retinal thickness and vascular imaging metrics ranges from poor to good, depending on population characteristics, comorbidities (e.g., glaucoma), imaging protocols, and specific metrics.^342^, ^343^, ^344^, ^345^, ^346^ Vessel density measures often show lower repeatability than thickness or perfusion, especially when comparing wide to narrow fields.^342^, ^347^

Most in vivo OCT studies feature sample sizes >100; however, *post mortem* and novel modality studies remain small. Nearly all studies exclude participants with ophthalmic or systemic conditions, such as diabetes (5% to 25% age‐standardized prevalence at 18 years)^348^ and glaucoma (1% to 5% prevalence under age 60 years),^349^ limiting generalizability. For example, in a UK Biobank study, 57% of individuals with both retinal OCT and brain MRI were excluded due to these conditions, with an additional 25% of the remainder removed for poor image quality, resulting in only ∼30% of the original cohort being analyzed.^3^

## 7 Datasets for research

Our team has completed the Ontario Neurodegenerative Disease Research Initiative (ONDRI, ClinicalTrials.gov ID NCT04104373), involving 520 individuals with neurodegenerative diseases. Participants were phenotyped using clinical data, structural and functional MRI, genomics, plasma biomarkers (e.g., Aβ and tau), and assessments of gait, speech, language, and eye tracking, among others. Retinal OCT retinal imaging was acquired in over half of the cohort. Follow‐up lasted 1 to 3 years for those with AD, Parkinson's disease, cerebrovascular disease, amyotrophic lateral sclerosis, and frontotemporal dementia.^350^, ^351^ The baseline data of this study was released in June 2022 (accessible at https://www.braincode.ca/content/controlled‐data‐releases). Additionally, the Brain Eye Amyloid Memory (BEAM) study (ClinicalTrials.gov ID NCT02524405) is under way, utilizing Aβ PET imaging and similar assessments and cohorts, as well as Lewy body disease.^310^ Other available cohorts include smaller preclinical AD cohorts (*n* < 300),^232^, ^233^ the Rotterdam study (*n* > 3000),^4^, ^352^ the UK Biobank (*n* > 65,000),^3^, ^353^, ^354^ and the Dunedin study (*n* > 800).^299^

Large‐scale data collection for OCT‐A studies is also under way in the Netherlands (Maastricht and Rotterdam studies), Germany (Rhineland study), the United States (Framingham study), and India (South Indian Genetics of Diabetic Retinopathy [SIGNATR] study).^355^

## 8 Future directions

Risk stratification and monitoring for adverse events, such as ARIA, are currently needed in anti‐amyloid therapy. Parallel to the brain exhibiting blood–brain barrier dysfunction and being prone to ARIA during anti‐amyloid therapy, the retina also exhibits vascular Aβ, compromised endothelial tight junctions,^128^ and neuroinflammation^118^ in AD. Combining promising retinal imaging modalities, such as OCTA to estimate small vessel disease (Section 5.2.3) and hyperspectral imaging to assess amyloid deposition (Section 5.2.6), may serve as a novel screening tool to identify those at risk of ARIA.

Furthermore, cell segmentation in higher‐resolution OCT using machine learning is promising. With retinal OCT images acquired at a cellular resolution, machine‐learning algorithms could segment and quantify subcellular structures, such as ganglion cell nuclei, which may be more informative than thickness, as retinal thinning can be masked by early AD processes.^26^ Our lab has developed a Bayesian 3D convolutional neural network with a U‐Net architecture for automatic segmentation and uncertainty estimation in the brain^173^; a similar solution could be applied to retinal segmentation. A significant challenge is obtaining large enough datasets to train generalizable models. Recent models in retinal imaging have been reviewed by Bahr et al.^356^

Additionally, underexplored brain regions, such as the basal forebrain, may correlate with ganglion cell nucleus count or GCL thickness due to their shared cholinergic properties. Finally, newer modalities such as choroidal thickness measurements, OCTA, electroretinogram, and hyperspectral imaging require further studies in preclinical AD and MCI to pool effect sizes in future meta‐analyses.

## 9 Conclusion

In summary, this review examines the relationship between AD pathology in the brain – particularly Aβ, tau, and vascular changes – and corresponding alterations in the retina. Evidence suggests abnormal Aβ and tau deposits in the retina may reflect brain pathology, although their forms may differ. Whether these deposits form through cell‐to‐cell transfer or *de novo* formation remains unclear. *Post mortem* studies indicate that retinal vascular changes may mirror brain pathology; however, data on other co‐pathologies are limited.

Retinal thickness changes, especially in layers with acetylcholine‐producing cells, are evident when comparing AD patients to controls, though this ability diminishes in early AD, consistent with recent meta‐analyses.^2^, ^322^ Correlations between retinal thickness and brain structures are also often weak when accounting for confounders, reducing diagnostic utility. Emerging higher‐resolution OCT techniques, however, may enhance diagnostic capabilities by quantifying cellular structures; however, physiological changes can mask gains from higher resolution.^357^, ^358^, ^359^

In contrast to retinal thickness measures, choroidal thickness shows promise, with larger effect sizes, as does vascular imaging, such as OCTA. However, studies on these methods are limited, and their effect sizes may decline somewhat when results are pooled and factors such as age, sex, and vascular risk factors are accounted for. We also discuss promising experimental retinal imaging techniques in AD, including hyperspectral image analysis for estimating retinal Aβ, and functional OCT methods. Further advancements in the most promising retinal biomarkers and their combination with other biomarkers may improve early AD screening and treatment monitoring.

## AUTHOR CONTRIBUTIONS

Conceptualization: MAB, SEB. Data curation: MAB, SHK, XJ, MK, JO. Formal analysis: MAB. Funding acquisition: MAB, SEB. Investigation: MAB, SHK, XJ, MK, JO, SEB, MG. Methodology: MAB, SHK, XJ, MK, SEB, MG. Project administration: MAB. Resources: MAB, SEB. Software: MAB. Supervision: SEB, MG, CH, PJK, RHS. Validation: MAB. Visualization (figures and tables): MAB, SHK, XJ, MK. Writing – original draft: MAB, SHK, XJ, MK. Writing – review & editing: MAB, SHK, XJ, MK, JO, RHS, PJK, CH, MG, SEB.

## CONFLICT OF INTEREST STATEMENT

MAB: None relevant to this manuscript. Graduate scholarships from Canadian Institutes of Health Research—Canadian Graduate Scholarship – Doctorate, Vision Science Research Program, Peterborough K.M. Hunter Graduate Scholarship, Queen Elizabeth II / Grace Lumsden and Margaret Nicholds, Graduate Scholarship in Science & Technology, Institute of Medical Science / University of Toronto Open Fellowship Award SCACE Graduate Fellowship in Alzheimer's Research, Ontario Graduate Scholarship (OGS) Award. SHK: None declared. XJ: None declared. MK: None declared. JO: None relevant to this manuscript. Grants from Alzheimer's Association & Brain Canada (24AARF‐1242638), BrightFocus (A2024012F), Alzheimer Society of Canada. RHS: None relevant to this manuscript. Funds from Ontario Brain Institute, Canadian Institutes of Health Research, National Institutes of Health. Payments made to institutions, not individuals. Follow MD Inc. (stock ownership). CH: None declared. PJK: None relevant to this manuscript. Payment or honoraria for lectures, presentations, speakers bureaus, manuscript writing or educational events from Amgen, Novartis, Bayer, Roche, RegenxBio, Apellis, Astellas. Participation on a Data Safety Monitoring Board or Advisory Board of Novartis, Bayer, Roche, Apellis, Astellas, Novelty Nobility, Johnson and Johnson, Kriya Therapeutics, AdMare Bioinnovations. Financial support (to institution) – Roche, Novartis, Bayer, RegenxBio, Johnson and Johnson, Neuracle Genetics. MG: None declared. SEB: None relevant to this manuscript. Grants or contract payments made to institution from contract research: Genentech, Optina, Roche, Eli Lilly, Eisa/Biogen Idec, NovoNordisk, Lilly Avid, ICON, Aribio Co., Maplight Therapeutics Peer Reviewed for Ontario Brain Institute, CIHR, Leducq Foundation, Heart and Stroke Foundation of Canada, NIH, Alzheimer's Drug Discovery Foundation, Brain Canada, Weston Brain Institute, Canadian Partnership for Stroke Recovery, Canadian Foundation for Innovation, Focused Ultrasound Foundation, Alzheimer's Association US, Queen's University, Compute Canada Resources for Research Groups, CANARIE, Networks of Centres of Excellence of Canada. Consulting fees paid to author or institution from Roche, Biogen, NovoNordisk, Eisai, Eli Lilly, DSR: Diagnosis, Solutions & Results Inc. Payment or honoraria for lectures, presentations, speakers bureaus, manuscript writing or educational events to author from Biogen, Roche New England Journal Manuscript, Roche Models of Care Analysis in Canada in Submission, Eisai MRI Workshop, Cpdnetwork planning committee for AD educational program. Participation on a Data Safety Monitoring Board or Advisory Board for Conference Board of Canada, World Dementia Council, University of Rochester Contribution to the Mission and Scientific Leadership of the Small Vessel VCID Biomarker Validation Consortium, National Institute of Neurological Disorders and Stroke, Ontario Dementia Care Alliance (ODCA). Author disclosures are available in the supporting information.

## Supporting information



Supporting Information

Supporting Information

## Data Availability

The authors confirm that the data supporting the findings of this study are available within the article and in references cited by the paper.
