# Supplementary material for: Brain and retina in Alzheimer's disease: Pathological intersections and estimates from imaging
Source: Alzheimers Dement. 2025 Nov 27;21(11):e70884. doi: 10.1002/alz.70884 (PMC12658388; doi:10.1002/alz.70884)
Supplement: Supplementary file 2 — Supporting Information [file ALZ-21-e70884-s001.docx]

**Supplements**

**Search Strategy Overview**

Our search strategy, informed by a proposed scale for assessing narrative reviews [[1](#_ENREF_1)], differs from systematic reviews by being more open-ended to synthesize knowledge across multiple subject areas. The majority of the search was completed by February 2023, with most updates by August 2025.

**Textbooks and Evidence-Based Resources:** We consulted textbooks for foundational knowledge on embryology, anatomy, and Alzheimer’s disease (AD) pathogenesis [[2](#_ENREF_2), [3](#_ENREF_3)]. We used evidence-based resources such as UpToDate [[4](#_ENREF_4)] for the latest insights on AD pathogenesis, clinical relevance, diagnosis, prognosis, treatments, and guidelines. We utilized Trip Pro and Epistemonikos databases for curated guidelines and evidence on retinal imaging in AD, as well as AD pathogenesis and diagnosis.

**Structured Search in Medline:** A structured search in Medline was conducted using keywords related to brain imaging and diagnostic categories, informed by previous meta-analyses [[5](#_ENREF_5)]. This search was not as comprehensive as a systematic review, employing specific keywords and filters to refine results. Keywords included but were not limited to: “Alzheimer disease” or “AD”, “retina”, “optical coherence tomography” or “OCT”, “OCT angiography”, “adaptive optics”, “hyperspectral imaging”, “magnetic resonance imaging” or “MRI”, “positron emission tomography” or “PET”, “amyloid”, “tau”, “synuclein”, and “TDP-43”.

**References and Future Citations:** We reviewed systematic review references [[5-12](#_ENREF_5)] for primary evidence not captured elsewhere. We used Scopus to track future citations of key papers, focusing on the most cited works, for example of retinal degeneration of AD [[13](#_ENREF_13)] or cholinergic brain MRI atlases [[14-17](#_ENREF_14)]. Furthermore, we used Google Scholar to find future citations of aforementioned papers; the search was usually limited to the five most cited papers listed.

**Researchers in the Field:** A pilot tool from Scopus helped identify relevant researchers since 2017 using keywords like "Alzheimer disease" AND "Brain" AND "Retina" AND "Tomography, Optical Coherence," scanning the top 50 authors' publications with results sorted by the highest matching document for studies.

**Clinical Trials:** Relevant clinical trials were identified on ClinicalTrials.gov by searching “Alzheimer disease” as “Condition or disease” and “Optical Coherence Tomography” as “Other terms”.

| **Table S1. Acetylcholine neurotransmitter location, function, and producing cells in the brain and retina.** | | | | | |
| --- | --- | --- | --- | --- | --- |
| **Location** | | **Function** | | **Production cell** | |
| **Brain** ^*^ | **Retina** | **Brain** | **Retina** | **Brain** ^*^ | **Retina** |
| BFCS, nbM ^†^ | INL/IPL border & GCL ^‡^ | Cognition (esp. attention and memory), ^§^ depth perception ^¶^ | Detection of directional motion ^#^ | Ch1-4 ^**^ | SAC ^††^ |
| Note.  ^*^While called a nucleus, the BFCS has more of an open structure, with cholinergically active cells dispersed among cells producing other neurotransmitters. Because of this open structure, anatomical boundaries do not necessarily define functional cell groups. The magnocellular cell groups that are cholinergically active in the basal forebrain are labelled as Ch1 to Ch4, with cholinergic cells in the nbM anatomical boundary labeled as Ch4 Liu et al., 2015 [[18](#_ENREF_18)].  ^†^Mesulam and Geula, 1988; Mesulam et al., 1983 [[19](#_ENREF_19), [20](#_ENREF_20)].  ^‡^Balasubramanian and Gan, 2014; Ford and Feller, 2012 [[21](#_ENREF_21), [22](#_ENREF_22)].  ^§^Ballinger et al., 2016; Hampel et al., 2019; Mesulam, 2013 [[23-25](#_ENREF_23)].  ^¶^Mendez et al., 1996 [[26](#_ENREF_26)].  ^#^Motion detection in the retina is largely regulated by GABA and acetylcholine Balasubramanian and Gan, 2014; Briggman et al., 2011; Ford and Feller, 2012; Taylor and Smith, 2012 [[21](#_ENREF_21), [22](#_ENREF_22), [27](#_ENREF_27), [28](#_ENREF_28)].  ^**^Liu et al., 2015; Mesulam and Geula, 1988; Mesulam et al., 1983 [[18-20](#_ENREF_18)].  ^††^Balasubramanian and Gan, 2014; Ford and Feller, 2012 [[21](#_ENREF_21), [22](#_ENREF_22)].  Abbreviations:  BFCS = basal forebrain cholinergic system, Ch 1-4 = cholinergic cell groups 1 to 4, GCL = ganglion cell layer, INL = inner nuclear layer, IPL = inner plexiform layer, nbM = nucleus basalis of Meynert, SAC = starburst amacrine cells. | | | | | |

| **Table S2.a. Significant associations in brain MRI and retinal optical coherence tomography in select studies with details.** | | | | | | | | | | |  |
| --- | --- | --- | --- | --- | --- | --- | --- | --- | --- | --- | --- |
|  | | **Grey matter (GM)** | | | | | | **GM+WM** | | |  |
|  | | Frontal | Temporal | Parietal | Occipital | BG-Th | Total | HP | | Total |  |
| Macula  Macula | GCC | 1 |  |  | 1 |  | 1 | 1 | | 1 |  |
|  | GCL |  | 2, 7^*^ | 2 | 3, 2 | 3 | 2 | 2 | |  |  |
|  | IPL |  | 2 | 2 | 2 |  | 2 |  | |  |  |
|  | GC-IPL |  | 8, 11 |  | 1, 8 |  | 1 | 12 | | 1, 12 |  |
|  | Fovea |  |  |  | 11 |  |  |  | |  |  |
|  | mRNFL |  |  |  |  | 4 | 1 | 1, 4 | | 1, 4 |  |
|  | mTotal | 1 | 7, 10 | 4, 9, 10 | 1, 4 | 4 | 1, 9, 10 | 1, 4 | | 1, 4 |  |
|  | pRNFL | 2 | 2, 5^*^, 7^*^, 8 |  | 3, 2, 5^*^, 11 | 3 | 2 | 2, 6, 5^*^ | |  |  |
|  |  | **White matter (WM)** | | | | | |  |  | | |
|  | GCC |  |  |  |  |  | 1 |  | |  |  |
|  | GCL | 2 | 2, 7^*^ | 2 | 2 |  | 2 |  | |  |  |
|  | IPL |  | 2 | 2 | 2 |  | 2 |  | |  |  |
|  | GC-IPL |  |  |  |  |  | 1 |  | |  |  |
|  | Fovea |  |  |  |  |  |  |  | |  |  |
|  | mRNFL |  |  |  |  | 4 |  |  | |  |  |
|  | mTotal |  | 7^*^ | 4 | 4 | 4 |  |  | |  |  |
|  | pRNFL | 2 | 2, 5^*^, 7^*^ |  | 2 | 12 | 2 | 12 | | 12 |  |
| Note.  Refer to **Supplementary Table 2b** for non-significant effects.  ^*^Indicates that grey and white matter were reported together and not separately.  Abbreviations:  BG-Th = Basal ganglia / thalamus (central or subcortical) brain region, GCC = Ganglion cell complex, GCL = Ganglion cell layer, GC-IPL = Ganglion cell-inner plexiform layers, GM = Grey matter, HP = Hippocampus, IPL = Inner plexiform layer, mRNFL = macula retina nerve fiber layer, pRNFL = peripapillary retina nerve fiber layer, WM = White matter.  References:  (1) Chua et al., 2021 [[29](#_ENREF_29)]: n = 2,131: UKBiobank. Adjusted for head size, age and sex. When adjusting for age, sex, and other factors the following significant associations become non-significant when compared to adjusting only for age and sex: mRNFL vs total brain, total GM, and hippocampus; mTotal vs. frontal lobe GM. These are listed both as significant and in non-significant associations for this reference.  (2) Mutlu et al., 2017 [[30](#_ENREF_30)]: n = 2,124: Adjusted for head size, age, sex and other factors.  (3) Mutlu et al., 2018 [[31](#_ENREF_31)]: n = 2,235: Rotterdam study. Adjusted for head size, age, sex, and other factors. Brain regions included cuneus, lingual, remaining occipital (grouped as occipital) and thalamus (BG-Th) assessed by voxel-based analysis. Associations were more likely significant with the left hemisphere for GCL.  (4) Sergott et al., 2021 [[32](#_ENREF_32)]: N varied by brain region = 656 to 1,111: Clinical trial NCT01739348. No adjustments. Baseline Pearson correlations included only: values for the hippocampus, thalamus, and parietal and occipital lobes are GM+WM. Mayo Cortical Thickness Index (entorhinal, inferior temporal, middle temporal, and fusiform as described by Dickerson et al., 2009 and Jack et al., 2015 [[33](#_ENREF_33), [34](#_ENREF_34)] was also associated with pRNFL but not mTotal. Only right thalamus and mTotal correlated longitudinally.  (5) Shi et al., 2020[[35](#_ENREF_35)]: n = 80: Adjusted for head size, age, sex and other factors. GM and WM reported together. Medial temporal results reported as temporal lobe in table, temporoparietal reported as parietal and midfrontal as frontal. Also included were the entorhinal, parahippocampal, lingual, pericalcarine, cuneus and lateral occipital regions, amongst which only lingual gyrus was significantly associated with pRNFL.  (6) Mendez-Gomez et al., 2018 [[36](#_ENREF_36)], n = 97: Three-City/Alienor studies. Adjusted for head size, age, sex and other factors.  (7) Casaletto et al., 2017 [[37](#_ENREF_37)]: n = 79: Adjusted for head size, age, sex and other factors. GM and WM reported together as such both GM and WM rows were marked as significant or non-significant for combined outcomes. Precuneus, posterior cingulate, primary motor cortex and basal ganglia were also reported but none were significant. Entorhinal significantly associated with macular volume, GCL volume and pRNFL. Total macula and GCL volume used instead of average thickness.  (8) Ong et al., 2015 [[38](#_ENREF_38)]: n = 164: Results adjusted for head size, age, sex, head size, axial length, OCT signal strength and vascular risk factors (mean arterial pressure, plasma blood glucose, serum cholesterol, and smoking). Central lobe classified as BG-Th in the table.  (9) den Haan et al., 2018 [[39](#_ENREF_39)]: n = 30: No adjustments, only Spearman correlation. Visual rating was used instead of automated measurements. Parietal and global cortical atrophy considered as GM only for this table.  (10) den Haan et al., 2019 [[40](#_ENREF_40)]: n = 134: Adjusted for age and sex, but not head size as visual ratings were used instead of automated measurements. Parietal and global cortical atrophy considered as GM for this table. Macula measure was perifoveal thickness not all sectors. Part of temporal lobe vs. perifoveal thickness would be significant without Bonferroni recalculation (*P* = .028), so is listed as significant to match other reporting.  (11) Mejia-Vergara et al., 2021 [[41](#_ENREF_41)]: n = 20: Adjusted for age and sex but not head size. Measurements were for pericalcarine (occipital lobe) and entorhinal (temporal lobe) grey matter, and total cerebellar volume. Fovea thickness was used instead of whole macula, as such only pRNFL is reported in the table. Association of pericalcarine (reported as occipital lobe in table) GM a GCL-IPL came close to statistical significance (*P* = 0.06) and is reported as non-significant. Association of entorhinal cortex is reported under temporal lobe. Total cerebellar volume was only significantly associated with GCL-IPL before Bonferroni recalculation and was not significantly associated with either pRNFL or fovea thickness. *P* values before multiple comparison calculation is reported in this table to be in line with reports from other studies.  (12) Barrett-Young et al., 2023 [[42](#_ENREF_42)]: n = 818 to 828: Adjusted for total brain volume, sex, and axial length. Measurements were for whole brain cortical surface area and thickness and ten subcortical regions amongst other measures. Cortical thickness is barely significant for GCL-IPL and not significant for pRNFL, as such both are marked as not significant. Cortical surface area and “brain age”, a score that aims to capture the discrepancy between a person’s chronological age and their estimated age based on multimodal imaging measures, were both significantly associated with retinal measures. Thalamus was significantly associated with pRNFL but not GCl-IPL. Other basal ganglia structures like caudate and putamen were either non-significant or marginally significant.  References with n > 100 and adjusted for age and sex: (1) Chua et al., 2021 [[29](#_ENREF_29)]; (2) Mutlu et al., 2017 [[30](#_ENREF_30)]; (3) Mutlu et al., 2018 [[31](#_ENREF_31)]; (4) Sergott et al., 2021 [[32](#_ENREF_32)]; (8) Ong et al., 2015 [[38](#_ENREF_38)]. | | | | | | | | | | |  |

| **Table S2.b. Brain MRI and retinal optical coherence tomography non-significant associations in select studies.** | | | | | | | | | | |  |
| --- | --- | --- | --- | --- | --- | --- | --- | --- | --- | --- | --- |
|  | | **Grey matter (GM)** | | | | | | **GM+WM** | | |  |
|  | | Frontal | Temporal | Parietal | Occipital | BG-Th | Total | HP | | Total |  |
| Macula  Macula | GCC |  | 1 |  |  |  |  |  | |  |  |
|  | GCL | 2, 7^*^ |  | 7^*^ |  |  |  |  | |  |  |
|  | IPL | 2 |  |  |  |  |  | 2 | |  |  |
|  | GC-IPL | 1, 8 | 1 | 8 | 11 | 8, 12 |  | 1 | | 12 |  |
|  | Fovea |  | 11 |  |  |  |  |  | |  |  |
|  | mRNFL | 1 | 1 | 4 | 1, 4 |  | 1 | 1 | | 1 |  |
|  | mTotal | 7^*^ | 1, 9 | 7^*^ |  |  |  |  | |  |  |
|  | pRNFL | 7^*^, 8 | 10, 11 | 2, 7^*^, 8, 10 | 8 | 7^*^, 8 | 6, 10 |  | | 6 |  |
|  |  | **White matter (WM)** | | | | | |  |  | | |
|  | GCC |  |  |  |  |  |  |  | |  |  |
|  | GCL | 7^*^ |  | 7^*^ |  |  |  |  | |  |  |
|  | IPL | 2 |  |  |  |  |  |  | |  |  |
|  | GC-IPL | 8 | 8 | 8 | 8 | 8, 12 |  |  | |  |  |
|  | Fovea |  |  |  |  |  |  |  | |  |  |
|  | mRNFL |  |  | 4 | 4 |  | 1 |  | |  |  |
|  | mTotal | 7^*^ |  | 7^*^ |  |  |  |  | |  |  |
|  | pRNFL | 7^*^, 8 | 8 | 2, 7^*^, 8 | 8 | 7^*^, 8, 12 | 6 |  | | 12 |  |
| Note.  Refer to **supplementary Table 2a** for details of each reference.  ^*^Indicates that grey and white matter were reported together and not separately.  Abbreviations:  BG-Th = Basal ganglia / thalamus (central or subcortical) brain region, GCC = Ganglion cell complex, GCL = Ganglion cell layer, GC-IPL = Ganglion cell-inner plexiform layers, GM = Grey matter, HP = Hippocampus, IPL = Inner plexiform layer, mRNFL = macula retina nerve fiber layer, pRNFL = peripapillary retina nerve fiber layer, WM = White matter.  References:  (1) Chua et al., 2021 [[29](#_ENREF_29)]: n = 2,131; (2) Mutlu et al., 2017 [[30](#_ENREF_30)]: n = 2,124 (not listed in table S2.b); (3) Mutlu et al., 2018 [[31](#_ENREF_31)]: n = 2,235; (4) Sergott et al., 2021 [[32](#_ENREF_32)]: N varied by brain region = 656 to 1,111; (5) Shi et al., 2020 [[35](#_ENREF_35)]: n = 80 (not listed in table S2.b); (6) Mendez-Gomez et al., 2018 [[36](#_ENREF_36)]: n = 97; (7) Casaletto et al., 2017 [[37](#_ENREF_37)]: n = 79; (8) Ong et al., 2015 [[38](#_ENREF_38)]: n = 164; (9) den Haan et al., 2018 [[39](#_ENREF_39)]: n = 30; (10) den Haan et al., 2019 [[40](#_ENREF_40)]: n = 134; (11) Mejia-Vergara et al., 2021[[41](#_ENREF_41)]: n = 20; (12) Barrett-Young et al., 2023 [[42](#_ENREF_42)]: n = 818 to 828.  References with n > 100 and adjusted for age and sex: (1) Chua et al., 2021 [[29](#_ENREF_29)]; (2) Mutlu et al., 2017 [[30](#_ENREF_30)] (not listed in table S2.b); (3) Mutlu, et al. 2018 [[31](#_ENREF_31)]; (4) Sergott et al., 2021 [[32](#_ENREF_32)]; (8) Ong et al., 2015 [[38](#_ENREF_38)]. | | | | | | | | | | |  |

| **Table S3. Effect size magnitude and direction when estimating whole brain or temporal lobe structures from retinal thickness in select cross-sectional studies with details.** | | | | |
| --- | --- | --- | --- | --- |
| **Reference** | **Whole brain** | **Temporal lobe** | **Adj.?** | **Analysis sample** |
| Chua et al., 2021 [[29](#_ENREF_29)] | mThickness: −0.08 (GM+WM-total, std coef)  mGCL-IPL: −0.08 (GM+WM-total, std coef) | mThickness: −0.04 (GM+WM-T, std coef)  mGCL-IPL: −0.03 (GM+WM-T, std coef)  mThickness: −0.05 (GM+WM-HP, std coef)  mGCLIPL: −0.03 (GM+WM-HP, std coef) | A, H, S, o | 2,131 |
| Mutlu et al., 2018 [[31](#_ENREF_31)] | mGCL, pRNFL: Positive association ^*^ | mGCL, pRNFL: Positive association ^*^ | A, H, S, o | 2,235 |
| Mutlu et al., 2017 [[30](#_ENREF_30)] | pRNFL: −0.031 (GM-total, std coef)  −0.034 (WM-total, std coef)  mGCL: −0.046 (GM-total, std coef)  −0.049 (WM-total, std coef) | pRNFL: −0.026 (GM-T, std coef)  −0.043 (WM-T, std coef)  −0.045 (HP, std coef)  mGCL: −0.045 (GM-T, std coef)  −0.061 (WM-T, std coef)  −0.043 (HP, std coef) | A, H, S, o | 2,124 |
| Sergott et al., 2021 [[32](#_ENREF_32)] | mThickness ~ GM+WM-total  Regression: 801.8 & 864.5 (left & right)^†^  Pearson corr.: 0.218 & 0.233 (left & right) | mThickness ~ HP  Regression: 4.8 & 6.1 (HP, left & right, nonstd coef)  Pearson corr.: 0.117 & 0.145 (HP, left & right) | No | 948 to 1,111 (varied by brain measure) |
| Mendez-Gomez et al., 2018 [[36](#_ENREF_36)] | pRNFL: 0.174 (GM+WM-total fraction per 10 $\mu$m retina, 95% CI close to zero. nonstd coef) | pRNFL: 0.013 (HP fraction per 10 $\mu$m retina, 95% CI close to zero. nonstd coef) | A, S, o | 90 (HP) to 97 (whole brain) |
| Shi et al., 2020 [[35](#_ENREF_35)] | - | Effects likely standardized regression coefficients, although not stated. Adjusted regression effects are larger than unadjusted Pearson r values, possibly indicating non-standardized regression coefficients.  pRNFL: 0.199 (MTL, std ? coef)  0.122 (MTL, Pearson r)  0.214 (HP, std ? coef)  0.124 (HP, Pearson r)  0.077 (Corti-entorhinal-fs, std ? coef)  0.056 (Corti-entorhinal-fs, Pearson r) | A, H, S, o | 80 |
| Casaletto et al., 2017 [[37](#_ENREF_37)] | - | An outlier study in that effect sizes are large despite accounting for age and head size.  pRNFL: 0.31 (MTL, std coef)  39.3 (MTL nonstd coef)  mVolume: 0.23 (MTL, std coef)  3013.6 (MTL, nonstd coef)  mGCLVolume: 0.30 (MTL std coef)  12,590.4 (MTL nonstd coef) | A, H, S, o | 75 |
| Ong et al., 2015 [[38](#_ENREF_38)] ^‡^ | - | Retinal measure ~ brain measure^c^  Effects not comparable to other studies as retinal measure is outcome not predictor.  pRNFL: −2.77 (GM+WM-T, std coef)  mGCL-IPL: −2.32 (GM+WM-T, std coef) | A, H, S, o | 164 |
| den Haan et al., 2018 [[39](#_ENREF_39)] | pRNFL: not provided just stated as not significant.  mNon-Fovea: −0.443 (GM+WM-total, Spearman’s *r*) | pRNFL: not provided just stated as not significant.  mNon-Fovea: not provided just stated as not significant. | No | 30 |
| den Haan et al., 2019 [[40](#_ENREF_40)] | pRNFL: 0.001 (Cort-total-visual rating, std coef)  mNon-Fovea: −0.314 (Cort-total-visual rating, std coef) | pRNFL: −0.111 (Cort-MTL-visual rating, std coef)  mNon-Fovea: −0.204 (Cort-MTL-visual rating, std coef) | A, S | 134 |
| Mejia-Vergara et al., 2021 [[41](#_ENREF_41)] | - | mGCL-IPL: 0.025 (Cort-entorhinal-fs, likely std coef ($\beta$), not stated in methods though)  pRNFL: 0.036 (Cort-pericalcarine-fs, likely std coef ($\beta$), not stated in methods though)  Cort-entorhinal-fs not reported for pRNFL | A, o | 20 |
| Barrett-Young et al., 2023 [[42](#_ENREF_42)] | All ages were 45.  Study could not assess impact of age.  pRNFL: 0.04 (Cort, std coef)  –0.08 (BrainAge) ^§^  0.14 (Cort Surf)  mGCL-IPL: 0.08 (Cort, std coef)  –0.09 (BrainAge) ^§^  0.09 (Cort Surf) | All ages were 45.  Study could not assess impact of age.  pRNFL: 0.10 (HP, std coef)  0.04 (Amygdala, std coef)  mGCL-IPL: 0.06 (HP, std coef)  0.02 (Amygdala, std coef) | H, S, o | 818 to 828  (varied by brain measure) |
| Note.  Effect sizes are typically smaller (by 10-fold at times) when accounting for head size and age for brain volumes and just age for cortical thickness in comparison to unadjusted regression coefficients or Pearson’s r. The direction of effect is not always the same in different studies, even when using similar predictors and outcomes, possibly indicating that effect sizes are close to zero (no effect).  ^*^Based on result description and positive t values of the VBM analysis output, no coefficients provided for regression models were based on brain volumes.  ^†^Micrometer retina change to mm^3^ brain change.  ^‡^It appears this study had retinal thickness as the outcome and brain as the predictor; therefore, it has larger standardized coefficients, which are not comparable to others that have brain measures as outcome.  ^§^BrainAge is a composite measure that estimates the gap between the chronological and biological age of a person’s brain based on measures such as cortical thickness, surface area, volume of subcortical grey matter, white matter, and cerebrospinal fluid volume [[43](#_ENREF_43)].  Abbreviations:  *Brain*: GM+WM = indicates sum of GM and WM, GM = Grey matter, WM = white matter, Cort = cortical thickness (freesurfer (fs) or visual rating), Cort Surf = cortical surface area, MTL = Medial temporal lobe, T = temporal, HP = hippocampus.  *Retina*: GCL = ganglion cell layer, IPL = inner plexiform layer, mNon-Fovea = para- and peri-fovea area (inner and outer macula on ETDRS grid), mThickness = total macula thickness, pRNFL = peripapillary retinal nerve fibre layer. The “p” prefix denotes peripapillary area and the “m” prefix denotes macula area. All measures are in thickness except where indicated as volume.  *Statistical*: A = age, Adj. = adjusted for potential confounders, H = head size in model as predictor or fraction of brain volume to head size used, o = other variables such as vascular risk factors, S = sex, std coef = standardized (regression coefficient). A question mark “?” indicates the value is likely a standardized coefficient but the paper was not clear. | | | | |

**References**

[1] Baethge C, Goldbeck-Wood S, Mertens S. SANRA-a scale for the quality assessment of narrative review articles. Res Integr Peer Rev. 2019;4:5.

[2] Martin JH. Neuroanatomy : text and atlas. 5th ed2020.

[3] Braak H, Del Tredici K. Neuroanatomy and pathology of sporadic Alzheimer's disease. Adv Anat Embryol Cell Biol. 2015;215:1–162.

[4] Keene CD, J MT, Kuller LH. Epidemiology, pathology, and pathogenesis of Alzheimer disease. In: Post TW, editor. UpToDate. Waltham, MA: UpToDate Inc.

[5] Chan VTT, Sun Z, Tang S, Chen LJ, Wong A, Tham CC, et al. Spectral-Domain OCT Measurements in Alzheimer's Disease: A Systematic Review and Meta-analysis. Ophthalmology. 2019;126:497–510.

[6] Coppola G, Di Renzo A, Ziccardi L, Martelli F, Fadda A, Manni G, et al. Optical Coherence Tomography in Alzheimer's Disease: A Meta-Analysis. PLoS One. 2015;10:e0134750.

[7] Ge YJ, Xu W, Ou YN, Qu Y, Ma YH, Huang YY, et al. Retinal biomarkers in Alzheimer's disease and mild cognitive impairment: A systematic review and meta-analysis. Ageing Res Rev. 2021;69:101361.

[8] Thomson KL, Yeo JM, Waddell B, Cameron JR, Pal S. A systematic review and meta-analysis of retinal nerve fiber layer change in dementia, using optical coherence tomography. Alzheimers Dement (Amst). 2015;1:136–43.

[9] Wang M, Zhu Y, Shi Z, Li C, Shen Y. Meta-analysis of the relationship of peripheral retinal nerve fiber layer thickness to Alzheimer's disease and mild cognitive impairment. Shanghai Arch Psychiatry. 2015;27:263–79.

[10] Mejia-Vergara AJ, Restrepo-Jimenez P, Pelak VS. Optical Coherence Tomography in Mild Cognitive Impairment: A Systematic Review and Meta-Analysis. Front Neurol. 2020;11:578698.

[11] Noah AM, Almghairbi D, Moppett IK. Optical coherence tomography in mild cognitive impairment - Systematic review and meta-analysis. Clin Neurol Neurosurg. 2020;196:106036.

[12] Knoll B, Simonett J, Volpe NJ, Farsiu S, Ward M, Rademaker A, et al. Retinal nerve fiber layer thickness in amnestic mild cognitive impairment: Case-control study and meta-analysis. Alzheimers Dement (Amst). 2016;4:85–93.

[13] Hinton DR, Sadun AA, Blanks JC, Miller CA. Optic-nerve degeneration in Alzheimer's disease. N Engl J Med. 1986;315:485–7.

[14] Teipel SJ, Flatz WH, Heinsen H, Bokde AL, Schoenberg SO, Stockel S, et al. Measurement of basal forebrain atrophy in Alzheimer's disease using MRI. Brain. 2005;128:2626–44.

[15] Zaborszky L, Hoemke L, Mohlberg H, Schleicher A, Amunts K, Zilles K. Stereotaxic probabilistic maps of the magnocellular cell groups in human basal forebrain. Neuroimage. 2008;42:1127–41.

[16] Kilimann I, Grothe M, Heinsen H, Alho EJ, Grinberg L, Amaro E, Jr., et al. Subregional basal forebrain atrophy in Alzheimer's disease: a multicenter study. J Alzheimers Dis. 2014;40:687–700.

[17] Fritz HJ, Ray N, Dyrba M, Sorg C, Teipel S, Grothe MJ. The corticotopic organization of the human basal forebrain as revealed by regionally selective functional connectivity profiles. Hum Brain Mapp. 2019;40:868–78.

[18] Liu AK, Chang RC, Pearce RK, Gentleman SM. Nucleus basalis of Meynert revisited: anatomy, history and differential involvement in Alzheimer's and Parkinson's disease. Acta Neuropathol. 2015;129:527–40.

[19] Mesulam MM, Geula C. Nucleus basalis (Ch4) and cortical cholinergic innervation in the human brain: observations based on the distribution of acetylcholinesterase and choline acetyltransferase. The Journal of comparative neurology. 1988;275:216–40.

[20] Mesulam MM, Mufson EJ, Levey AI, Wainer BH. Cholinergic innervation of cortex by the basal forebrain: cytochemistry and cortical connections of the septal area, diagonal band nuclei, nucleus basalis (substantia innominata), and hypothalamus in the rhesus monkey. The Journal of comparative neurology. 1983;214:170–97.

[21] Ford KJ, Feller MB. Assembly and disassembly of a retinal cholinergic network. Vis Neurosci. 2012;29:61–71.

[22] Balasubramanian R, Gan L. Development of Retinal Amacrine Cells and Their Dendritic Stratification. Curr Ophthalmol Rep. 2014;2:100–6.

[23] Mesulam MM. Cholinergic circuitry of the human nucleus basalis and its fate in Alzheimer's disease. The Journal of comparative neurology. 2013;521:4124–44.

[24] Hampel H, Mesulam MM, Cuello AC, Khachaturian AS, Vergallo A, Farlow MR, et al. Revisiting the Cholinergic Hypothesis in Alzheimer's Disease: Emerging Evidence from Translational and Clinical Research. J Prev Alzheimers Dis. 2019;6:2–15.

[25] Ballinger EC, Ananth M, Talmage DA, Role LW. Basal Forebrain Cholinergic Circuits and Signaling in Cognition and Cognitive Decline. Neuron. 2016;91:1199–218.

[26] Mendez MF, Cherrier MM, Meadows RS. Depth perception in Alzheimer's disease. Percept Mot Skills. 1996;83:987–95.

[27] Taylor WR, Smith RG. The role of starburst amacrine cells in visual signal processing. Vis Neurosci. 2012;29:73–81.

[28] Briggman KL, Helmstaedter M, Denk W. Wiring specificity in the direction-selectivity circuit of the retina. Nature. 2011;471:183–8.

[29] Chua SYL, Lascaratos G, Atan D, Zhang B, Reisman C, Khaw PT, et al. Relationships between retinal layer thickness and brain volumes in the UK Biobank cohort. Eur J Neurol. 2021;28:1490–8.

[30] Mutlu U, Bonnemaijer PWM, Ikram MA, Colijn JM, Cremers LGM, Buitendijk GHS, et al. Retinal neurodegeneration and brain MRI markers: the Rotterdam Study. Neurobiol Aging. 2017;60:183–91.

[31] Mutlu U, Ikram MK, Roshchupkin GV, Bonnemaijer PWM, Colijn JM, Vingerling JR, et al. Thinner retinal layers are associated with changes in the visual pathway: A population-based study. Hum Brain Mapp. 2018;39:4290–301.

[32] Sergott RC, Raji A, Kost J, Sur C, Jackson S, Locco A, et al. Retinal Optical Coherence Tomography Metrics Are Unchanged in Verubecestat Alzheimer's Disease Clinical Trial but Correlate with Baseline Regional Brain Atrophy. J Alzheimers Dis. 2021;79:275–87.

[33] Jack CR, Jr., Wiste HJ, Weigand SD, Knopman DS, Mielke MM, Vemuri P, et al. Different definitions of neurodegeneration produce similar amyloid/neurodegeneration biomarker group findings. Brain. 2015;138:3747–59.

[34] Dickerson BC, Bakkour A, Salat DH, Feczko E, Pacheco J, Greve DN, et al. The cortical signature of Alzheimer's disease: regionally specific cortical thinning relates to symptom severity in very mild to mild AD dementia and is detectable in asymptomatic amyloid-positive individuals. Cereb Cortex. 2009;19:497–510.

[35] Shi Z, Cao X, Hu J, Jiang L, Mei X, Zheng H, et al. Retinal nerve fiber layer thickness is associated with hippocampus and lingual gyrus volumes in nondemented older adults. Prog Neuropsychopharmacol Biol Psychiatry. 2020;99:109824.

[36] Mendez-Gomez JL, Pelletier A, Rougier MB, Korobelnik JF, Schweitzer C, Delyfer MN, et al. Association of Retinal Nerve Fiber Layer Thickness With Brain Alterations in the Visual and Limbic Networks in Elderly Adults Without Dementia. JAMA Netw Open. 2018;1:e184406.

[37] Casaletto KB, Ward ME, Baker NS, Bettcher BM, Gelfand JM, Li Y, et al. Retinal thinning is uniquely associated with medial temporal lobe atrophy in neurologically normal older adults. Neurobiol Aging. 2017;51:141–7.

[38] Ong YT, Hilal S, Cheung CY, Venketasubramanian N, Niessen WJ, Vrooman H, et al. Retinal neurodegeneration on optical coherence tomography and cerebral atrophy. Neurosci Lett. 2015;584:12–6.

[39] den Haan J, Janssen SF, van de Kreeke JA, Scheltens P, Verbraak FD, Bouwman FH. Retinal thickness correlates with parietal cortical atrophy in early-onset Alzheimer's disease and controls. Alzheimers Dement (Amst). 2018;10:49–55.

[40] den Haan J, van de Kreeke JA, Konijnenberg E, ten Kate M, den Braber A, Barkhof F, et al. Retinal thickness as a potential biomarker in patients with amyloid-proven early- and late-onset Alzheimer's disease. Alzheimers Dement (Amst). 2019;11:463–71.

[41] Mejia-Vergara AJ, Karanjia R, Sadun AA. OCT parameters of the optic nerve head and the retina as surrogate markers of brain volume in a normal population, a pilot study. J Neurol Sci. 2021;420:117213.

[42] Barrett-Young A, Abraham WC, Cheung CY, Gale J, Hogan S, Ireland D, et al. Associations Between Thinner Retinal Neuronal Layers and Suboptimal Brain Structural Integrity in a Middle-Aged Cohort. Eye Brain. 2023;15:25–35.

[43] Liem F, Varoquaux G, Kynast J, Beyer F, Kharabian Masouleh S, Huntenburg JM, et al. Predicting brain-age from multimodal imaging data captures cognitive impairment. Neuroimage. 2017;148:179–88.
